# RNA-independent cis-autoregulatory circuits within bidirectional gene pairs control metabolism

**DOI:** 10.1126/sciadv.aei6204

**Published:** 2026-09-11

**Authors:** Xiang-Yu Liu, Yingying Yu, Guo-Xiao Wang, Ruben Garcia-Martin, C. Ronald Kahn

**Affiliations:** ^1^ Section of Integrative Physiology and Metabolism, Joslin Diabetes Center, and Department of Medicine, Harvard Medical School, Boston, MA 02115, USA.; ^2^ Broad Institute of MIT and Harvard, Cambridge, MA 02142, USA.

## Abstract

Many genes share promoters with another gene on the opposite DNA strand, forming bidirectional pairs. During metabolic transitions, most pairs are coordinately regulated in the liver, in part through chromatin topology. In pairs composed of a long noncoding RNA (lncRNA) and a protein-coding gene (PCG), lncRNA transcription cis-activates PCG transcription by establishing an active chromatin environment at the promoter-proximal enhancer of PCG. Reciprocally, PCG transcription cis-inhibits lncRNA transcription, forming internal cis-autoregulatory circuits. Both directions of regulation are independent of the RNA products but dependent on the transcription process itself. Similar promoter-proximal enhancer signatures and cis-autoregulatory circuits are present in many lncRNA/PCG and PCG/PCG pairs, underscoring the generalizability of these circuits. Using the conserved lncRNA/PCG pair *Gm15663*/*Tmtc2* as a model, we show that blocking lncRNA transcription in the liver in vivo disrupts this coordinated regulation, decreasing *Tmtc2* expression and inducing hepatic steatosis. These data demonstrate the physiological importance of transcription-dependent cis-autoregulatory circuits in metabolic homeostasis and their potential for dysregulation in disease.

## INTRODUCTION

The liver is central to metabolic homeostasis, balancing systemic energy needs by storing and mobilizing nutrients (*1*). These processes are largely driven by a balance in the actions of insulin and glucagon and their opposing regulatory effects on gene transcription (*2*–*8*). Key metabolic regulators such as glucose-6-phosphatase (*G6pc*) and lipin-1 (*Lpin1*) are antagonistically regulated by insulin and glucagon and their associated metabolic conditions, altering glucose and lipid metabolism (*2*, *5*, *9*, *10*). In addition to these protein-coding genes (PCGs), the mammalian genome includes a large number of long noncoding RNAs (lncRNAs), i.e., transcripts longer than 200 nucleotides that do not encode peptides (*11*–*13*). While less studied than PCGs, lncRNAs can also be dynamically regulated by hormones and metabolic diseases (*14*–*17*). It has been reported that some metabolically regulated lncRNAs affect glucose and lipid metabolism through interacting with key metabolic pathways, such as SREBP1c and FXR (*14*, *15*). Despite these advances, the precise contributions of most lncRNAs to hepatic metabolic adaptation remain poorly defined. This gap is compounded by the substantial variability in lncRNA sequences across species, making it challenging to generalize findings and fully understand their roles in metabolism (*18*, *19*).

A significant fraction of conserved lncRNA genes and PCGs are adjacent to other PCGs and transcribed from the same promoter but off the opposite DNA strand and in the opposite direction (*20*). These lncRNAs and their adjacent PCGs, as well as two adjacent PCGs, are known as bidirectional lncRNA/PCG pairs or PCG/PCG pairs (*11*, *21*), which have been described in mammals and other species (*20*, *22*–*24*). Gene Ontology (GO) pathway analysis showed that bidirectional lncRNAs or PCGs are involved in many processes, such as lipid metabolism and transcription regulation (fig. S1A). Some lncRNAs have been shown to cis-regulate their neighboring PCGs in states of chronic regulation, particularly in the context of development and tumor (*25*–*28*). Mechanistically, most studies have focused on their roles mediated by lncRNA transcripts through their interactions with protein, RNA, or DNA (*15*, *19*, *29*–*34*). On the other hand, emerging evidence suggests that the lncRNA transcription activity itself may play a significant role in cis-regulation independent of RNA transcripts (*35*). This involves the modulation of permissive chromatin environment and regulatory elements (*27*, *36*). However, functions of lncRNA mediated by the transcription process itself are less studied than those mediated by lncRNA transcripts largely due to the difficulty in isolating the effects of transcription per se from those of resultant RNA transcripts (*37*, *38*). Furthermore, whether these types of cis-regulatory mechanisms play any role in more short-term gene regulation, such as across metabolically regulated bidirectional lncRNAs, is less clear.

Here, we show that during metabolic homeostasis, most bidirectional genes in liver show coordinated regulation. These include both lncRNA/PCG pairs and PCG/PCG pairs. Among these regulated pairs, we have identified an evolutionally conserved lncRNA/PCG pair, *Gm15663*/*Tmtc2* (transmembrane *O*-mannosyltransferase targeting cadherins 2), and we used this pair as a model to investigate how such closely positioned genes influence each other and how this interplay controls metabolism. Mechanistically, using multiple orthogonal methods, we systematically demonstrate that RNA-independent reciprocal cis-autoregulatory circuits mediated by transcription itself between *Gm15663* and *Tmtc2* play a crucial role. These circuits are also pivotal in controlling other metabolically regulated lncRNA/PCG pairs, and even PCG/PCG pairs, to maintain metabolic homeostasis. Inhibiting *Gm15663* transcription in the liver induced hepatic steatosis and abnormal systemic lipid distribution by decreasing *Tmtc2* levels. Thus, by uncovering cis-autoregulatory circuits as a hormone-responsive mechanism embedded within bidirectional gene pairs, our work adds a previously unrecognized dimension to the understanding of hormone-regulated metabolism. Our study also indicates that targeting these transcription-mediated regulatory circuits could provide novel therapeutic approaches for intervention in type 2 diabetes and metabolic dysfunction-associated steatotic liver disease.

## RESULTS

### Coordinated regulation of bidirectional gene pairs by insulin, glucagon, and their associated metabolic conditions

**Fig. 1.**
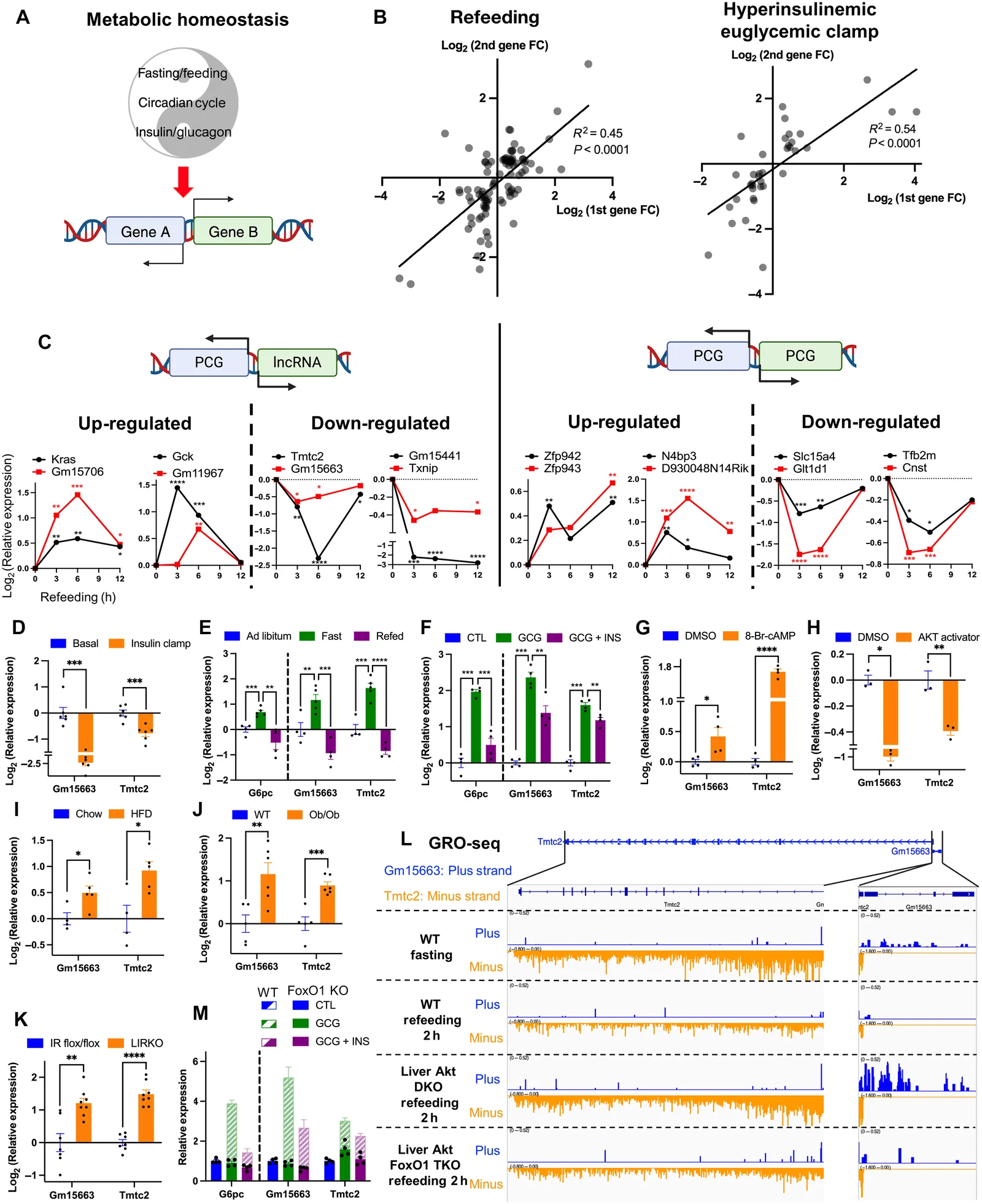
*Gm15663*/*Tmtc2* transcription regulation under metabolic conditions. (**A**) Overview of the regulation of bidirectional gene pairs by multiple metabolic conditions. Created in BioRender. X. Liu. (2026) https://BioRender.com/3sygsij. (**B**) Regulation of bidirectional gene pairs in mouse liver at 12 hours after refeeding compared to fasting and during hyperinsulinemic euglycemic clamp. Linear regression analysis of change on a log_2_ scale. RNA sequencing (RNA-seq) of fasting/feeding transition are from the GSE137385 dataset (male C57BL6/J mice on low-fat diet, *n* = 3 to 5). RNA-seq of hyperinsulinemic euglycemic clamp are from GSE117741 (liver samples). (**C**) Transcription regulatory kinetics of exemplary lncRNA/PCG and PCG/PCG pairs in mouse liver at fasting and at 3, 6, and 12 hours after refeeding. Significance and *P* values were determined using DESeq2. Created in BioRender. X. Liu. (2026) https://BioRender.com/3sygsij. (**D** and **E**) Regulation of *Gm15663*/*Tmtc2* in livers of mice after insulin clamp (D) or after overnight fasting and 6-hour refeeding (E). [(D) *n =* 6, (E) *n =* 4 to 5]. (**F**) Regulation of the *Gm15663*/*Tmtc2* by 100 nM glucagon (GCG) without and with 100 nM insulin (INS) in primary hepatocytes from C57BL6/J mice (6-hour stimulation). (*n =* 4). (**G** and **H**) Regulation of the *Gm15663*/*Tmtc2* gene pair in mouse primary hepatocytes following 6-hour stimulation by 20 μM 8-Br-cAMP (G) or 10 μM SC79, an AKT chemical activator (H). [(G) *n =* 4, (H) *n =* 3]. (**I** to **K**) Relative expression of the *Gm15663*/*Tmtc2* in the liver of C57BL6/J male mice after 12 weeks of HFD (I), 10-week-old ob/ob mice (J), and 12-week-old mice with liver-specific insulin receptor knockout (LIRKO) (K). [(I) *n =* 4 to 5, (J) *n =* 5 to 6, (K) *n =* 7 to 8]. (**L**) Global run-on sequencing (GRO-seq) datasets on liver samples of wild-type (WT)–fasting, WT-refeeding, liver-Akt-DKO–refeeding, and liver-Akt-Foxo1-TKO–refeeding mice (from GSE189810). (**M**) Relative expression of *Gm15663*/*Tmtc2* in primary hepatocytes from mice with liver-specific *FoxO1* knockout (solid fill) and WT mice (striped fill). (*n =* 4). h, hours.

To identify potential commonalities in the metabolic regulation of bidirectional gene pairs in the liver, we analyzed the transcriptome in response to fasting and feeding (*39*), throughout a circadian cycle (*40*), and after elevating insulin levels in a hyperinsulinemic euglycemic clamp (Fig. 1A) (*16*). Here, bidirectional gene pairs were defined as two genes on opposite DNA strands whose annotated transcription start sites (TSSs) are separated by less than 2 kb; coregulated pairs were defined as pairs in which both genes were significantly regulated. In general, consistent with previous studies in other systems for gene regulation (*22*, *41*), bidirectional gene pairs exhibited highly coordinated regulation in the liver, as shown by the correlated changes in gene expression during fasting and following refeeding at 3, 6, and 12 hours (Fig. 1B and fig. S1, B to G) and after insulin clamp (Fig. 1B and fig. S1H). These coordinately regulated gene pairs (listed in tables S1 to S4) contain numerous PCGs important for metabolic homeostasis. Among these gene pairs, 17 pairs were regulated in the same direction in liver both in the insulin clamp and during refeeding. In these 17 pairs, while some pairs consisted of two PCGs, 9 of these pairs were lncRNA/PCG pairs (fig. S2A). These gene pairs exhibited coordinated regulatory kinetics during their response to refeeding (Fig. 1C and fig. S2B) and during both the light and dark periods (figs. S2, C and D; examples shown in fig. S2E) (*40*). The PCG pairs included many important metabolic regulators, such as the amino acid transporter *Slc15a4* and acetyl–coenzyme A acyltransferase 1A (*Acaa1a*) involved in β-oxidation. The PCGs paired with lncRNAs also included many crucial genes involved in metabolism including *Txnip*, *Kras*, and *Gck*.

Among these metabolically coordinated bidirectional pairs, we identified *Gm15663*/*Tmtc2*, a pair exhibiting significant conservation in both relative genomic positioning and gene sequence between mouse and human (fig. S3, A and B). *Gm15663* was also the most insulin–down-regulated lncRNA, while *Tmtc2* plays a crucial role in endoplasmic reticulum (ER) homeostasis (*42*) and has been implicated in type 2 diabetes pathogenesis by human genome-wide association studies (*43*, *44*). Thus, we initially used *Gm15663*/*Tmtc2* as a model to investigate how two closely positioned genes in a bidirectional pair influence each other under metabolic conditions and how this process controls hepatic metabolism, and we subsequently examined other pairs to assess the generalizability of these findings.

### The conserved *Gm15663*/*Tmtc2* lncRNA/PCG pair is coordinately regulated in multiple metabolic conditions

Using 5′ rapid amplification of cDNA ends (5′ RACE) followed by complete amplicon sequencing, we determined that the TSSs of *Gm15663* and *Tmtc2* were only 80 base pairs apart (fig. S3, C to E). Both *Gm15663* and *Tmtc2* were coordinately down-regulated during insulin clamp (Fig. 1D). Consistent with this, up-regulation of *Gm15663*/*Tmtc2* by fasting and down-regulation by refeeding in the liver could be confirmed using quantitative reverse transcription polymerase chain reaction (qRT-PCR), mirroring the regulatory pattern of *G6pc* (Fig. 1E). Both *Gm15663* and *Tmtc2* also exhibited a synchronous oscillation with the physiological circadian rhythm in mouse liver, with increases during the light cycle (physiological fasting) and decreases during the dark cycle (physiological feeding) (fig. S2E).

Glucagon and insulin are two major antagonistic drivers of metabolic regulation. During fasting and its associated circadian period, glucagon levels are high and insulin levels are low, and the converse is true during feeding. These two hormones also play an antagonistic effect on gene regulation in the liver. Using isolated primary mouse hepatocytes, we confirmed that *Gm15663* and *Tmtc2* were also coordinately regulated, with an increase in expression following glucagon treatment, and this up-regulation was reduced by insulin (Fig. 1F). It is known that the actions of glucagon are largely mediated by adenosine 3′,5′-monophosphate (cAMP)/protein kinase A axis, while the metabolic actions of insulin are largely mediated by the phosphoinositide 3-kinase/AKT axis (*45*, *46*). Consistent with this, in isolated hepatocytes, 8-Br-cAMP up-regulated *Gm15663* and *Tmtc2* (Fig. 1G), while a chemical AKT activator SC79 down-regulated this gene pair (Fig. 1H). The antagonistic effects by glucagon and insulin on this gene pair also extended to their pathophysiological regulation. In states of insulin resistance, such as high-fat diet–induced obesity and ob/ob mice, the effects of glucagon predominate over insulin, and both *Gm15663* and *Tmtc2* exhibited up-regulation in the liver (Fig. 1, I and J). Likewise, this gene pair was also up-regulated in the liver with genetically driven insulin resistance due to liver-specific knockout of insulin receptor (Fig. 1K). Thus, together, these data show coordinated regulation of lncRNA *Gm15663* and its neighboring PCG *Tmtc2*, under the control of insulin, glucagon, and multiple metabolic conditions.

Treatment of mouse primary hepatocytes with triptolide, an inhibitor of transcription, completely blocked the regulation of *G6pc* and *Gm15663*/*Tmtc2* by glucagon and insulin (fig. S3F). These data indicate that these two hormones regulate the *Gm15663*/*Tmtc2* pair exclusively through transcription, similar to *G6pc*. It has been reported that stronger nucleosome positioning surrounding TSS is associated with increased gene expression (*47*–*49*). Thus, we used micrococcal nuclease to digest the chromatin to mono-nucleosomes followed by qPCR (fig. S3G) (*50*–*52*). The data showed that glucagon stimulation increased the signals of mononucleosome around the *Gm15663*/*Tmtc2* promoter, indicating stronger nucleosome positioning and transcriptional regulation by glucagon. In addition, analysis of RNA polymerase II (Pol II) chromatin immunoprecipitation sequencing (ChIP-seq) on liver samples through an entire circadian cycle showed that Pol II occupancy around the *Gm15663*/*Tmtc2* promoter correlated with their expression and circadian rhythm (figs. S3H and S2E) (*53*), further indicating that *Gm15663*/*Tmtc2* are coregulated at the transcriptional level.

### FOXO1 and chromatin topology mediate transcriptional regulation on *Gm15663*/*Tmtc2* in metabolism

The transcription factor (TF) FOXO1 has been shown to play an important role in the antagonistic regulation of gene transcription by both glucagon and insulin, serving as the downstream of both the cAMP and AKT pathways (*2*, *10*). Notably, both pathways have been shown to regulate *Gm15663*/*Tmtc2* (see Fig. 1, G and H). FOXO1 translocation into the nucleus following glucagon stimulation and its retention in the cytoplasm following insulin simulation underscore its regulatory effects in metabolism (*2*, *54*). Thus, we hypothesize that FOXO1 may mediate the regulation of *Gm15663*/*Tmtc2* by glucagon and insulin. Analysis of Global Run-On Sequencing data from liver-specific *Akt1*/*2*–double-knockout (*Akt*-DKO) and *FoxO1*-triple-knockout (*Akt*-*FoxO1*-TKO) mice (*55*) demonstrated an increase in transcription of *Gm15663*/*Tmtc2* in *Akt*-DKO mice and a decrease in *Akt*-*FoxO1*-TKO mice, indicating FOXO1 as a crucial regulator of this gene pair (Fig. 1L). Moreover, hepatocytes from liver-specific *FoxO1* knockout mice showed blunted regulation of this gene pair by both hormones and related pathways (Fig. 1M and fig. S4, A and B), confirming the pivotal role of FOXO1 in their regulation.

**Fig. 2.**
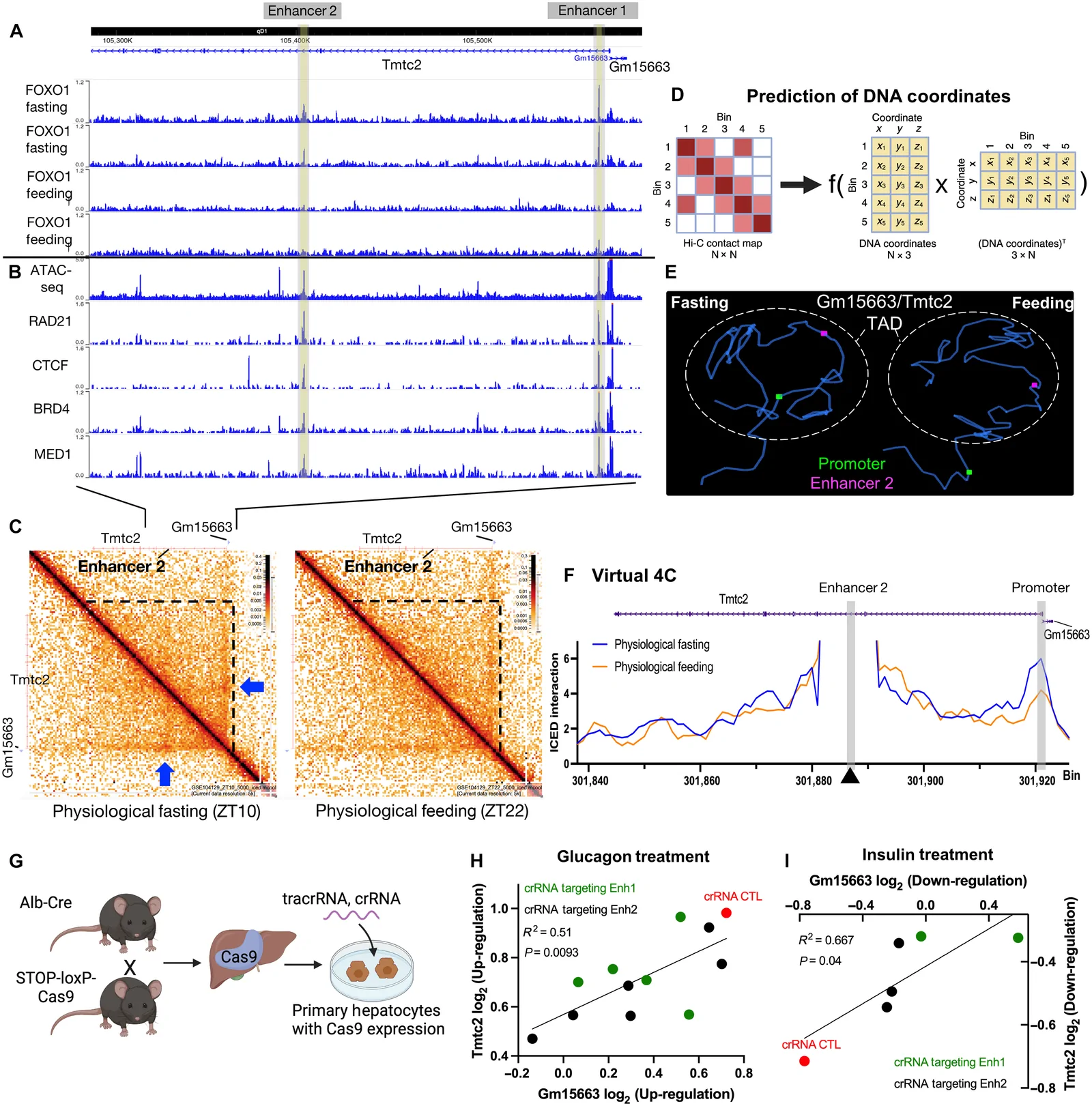
*Gm15663*/*Tmtc2* transcription regulation by FOXO1 and its associated chromatin topological changes. (**A**) FOXO1 ChIP-seq data in the region of *Gm15663*/*Tmtc2* genes in the livers of mice subjected to 12-hour fasting or 2-hour refeeding. ChIP-seq datasets are from GSE119713. (**B**) ATAC-seq and ChIP-seqs of cohesin (RAD21), CTCF, BRD4, and MED1 in the liver. See Materials and Methods for the origins of ChIP-seq datasets and other details. (**C**) Hi-C contact maps at ZT10 (fasting) and ZT22 (feeding) in the liver. Hi-C datasets were from GSE104129 (male C57BL6/J mice). Blue arrows are showing the interaction between Enh2 and promoter region of *Gm15663*/*Tmtc2*. (**D** and **E**) Reconstruction of simulated spatial chromatin organization of *Gm15663*/*Tmtc2* using FLAMINGO at 5-kb resolution. Green: promoter; purple: Enhancer 2. Created in BioRender. X. Liu (2026) https://BioRender.com/3sygsij. (**F**) Virtual 4C analysis of interactions between Enh2 and the whole *Gm15663*/*Tmtc2* TAD, including regions around the *Gm15663*/*Tmtc2* promoter. (**G**) Schematic showing generation of mice with hepatocyte-specific Cas9 expression and transfection of crRNA and tracrRNA. Created in BioRender. X. Liu (2026) https://BioRender.com/3sygsij. (**H** and **I**) Regulation of *Gm15663* and *Tmtc2* by glucagon (H) or insulin (I) after enhancer disruption using CRISPR-Cas9 in primary hepatocytes. Each dot represents the mean effect of one individual crRNA targeting Enh1 or Enh2, with biological replicates shown and statistically analyzed in fig. S5 (C and D). Red: negative control (CTL) crRNA. Green: crRNAs targeting Enh1. Black: crRNAs targeting Enh2. Glucagon-induced up-regulation and insulin-induced down-regulation of *Gm15663* and *Tmtc2* were plotted with linear regression analysis on a log_2_ scale. Glucagon treatment: 100 nM for 6 hours; insulin treatment: 100 nM for 6 hours. Expression was assessed by qRT-PCR.

Despite the clear role of FOXO1 in the regulation of this gene pair, analysis of the −2 to +2 kb promoter region of *Gm15663*/*Tmtc2* revealed no FOXO1-binding sequence [5′-(C/A)(A/C)AAA(C/T)AA-3′]. Instead, FOXO1 ChIP-seq from liver samples (*56*) under fasting (high glucagon) versus feeding (high insulin) conditions revealed two binding sites within *Tmtc2* introns, present only during fasting (Fig. 2A). These two FOXO1-binding sites were distal to *Gm15663*/*Tmtc2* promoter: ∼6.5 and ∼170 kb from Tmtc2 TSS. To systematically identify chromatin features associated with these two FOXO1-binding regions, we used the Cistrome Toolkit algorithm, which interrogates publicly available ChIP-seq datasets in the Cistrome database (*57*, *58*). For each region of interest, this pipeline identifies all histone posttranslational modification (PTM) ChIP-seq datasets in the database that contain peak signals at the specified genomic region (fig. S4C). The results showed that most mouse histone PTM ChIP-seq datasets with peak signals in these two FOXO1-binding regions are primarily H3K4me1/2/3 and H3K27Ac ChIP-seqs from liver samples (fig. S4D), suggesting that these two regions likely function as liver-specific active enhancers (fig. S4D). ChIP-seq data in the liver of other active enhancer chromatin signatures, e.g., BRD4 and MED1, also showed peak signals in these two regions (Fig. 2B). Likewise, analysis of ATAC-seqs from various tissues and cell types showed that only liver, but not other tissues, exhibited signals at these two FOXO1-binding regions (Fig. 2B and fig. S4E), confirming that these two FOXO1-binding regions likely serve as liver specific enhancers for *Gm15663*/*Tmtc2*. We named them enhancer 1 (Enh1) and 2 (Enh2) (Fig. 2, A and B), according to their respective positions downstream of the *Gm15663*/*Tmtc2* promoter (Enh1 at 6.5 kb and Enh2 at 170 kb).

To investigate the functional relationship between these two distal enhancers and *Gm15663*/*Tmtc2* promoter in liver, we defined the three-dimensional (3D) chromatin conformation by the analysis of high-throughput chromosome conformation capture (Hi-C) data on liver samples from physiological fasting/feeding mice during a normal circadian cycle (ZT10/ZT22) (*7*). Hi-C contact maps identified a topologically associated domain (TAD) containing only the *Gm15663* and *Tmtc2* genes, with increased Enh2-promoter looping at fasting, which decreased during feeding (Fig. 2C and fig. S5A). To reconstruct the spatial chromatin organization of *Gm15663* and *Tmtc2*, we used FLAMINGO to simulate DNA coordinates from Hi-C contact maps through low-rank matrix completion (Fig. 2D) (*59*). This analysis showed that during fasting, when their expression levels were elevated, the promoter of *Gm15663*/*Tmtc2* exhibited increased contact frequency with the TAD and Enh2 (Fig. 2E, fig. S5B, and movies S1 and S2). Analysis of the contact frequency focusing at the Enh2 across the TAD using virtual circularized chromosome conformation capture (virtual 4C) also showed more frequent contacts between Enh2 and the promoter during fasting (Fig. 2F), confirming their role in gene regulation.

It has been shown that CTCF and cohesin control chromatin organization and can mediate enhancer-promoter looping interaction (*60*). Analysis of CTCF and cohesin (RAD21 subunit) ChIP-seq data in mouse liver (*7*) showed that both proteins bound to both enhancer regions (Enh1 and Enh2) and the *Gm15663*/*Tmtc2* promoter (Fig. 2B). To confirm that the Enh1 and Enh2 were functional enhancers regulating *Gm15663*/*Tmtc2*, we generated mice with liver-specific overexpression of Cas9, isolated their hepatocytes, and disrupted Enh1 or Enh2 using targeting crRNAs to test whether each enhancer individually contributes to hormone-responsive regulation of the *Gm15663*/*Tmtc2* pair in primary hepatocytes (Fig. 2G). qRT-PCR revealed that disrupting either Enh1 or Enh2 significantly impaired glucagon-induced up-regulation of *Gm15663* and *Tmtc2* in a correlated manner across various crRNAs (Fig. 2H and fig. S5C). In contrast, glucagon-induced up-regulation of the *G6pc*, located in a different TAD, was unaffected by crRNAs targeting Enh1/Enh2 (fig. S5C). Similarly, disruption of Enh1 or Enh2 also significantly impaired the insulin-induced down-regulation of both *Gm15663* and *Tmtc2*, but not *G6pc* (Fig. 2I and fig. S5D). Together, these data show that *FoxO1* mediates the regulation of *Gm15663*/*Tmtc2* by glucagon and insulin by binding to *Tmtc2*-intronic enhancers and facilitating their topological looping with the promoter (fig. S5E).

### RNA-independent cis-autoregulatory circuits between *Gm15663* and *Tmtc2* are mediated by transcription process itself

**Fig. 3.**
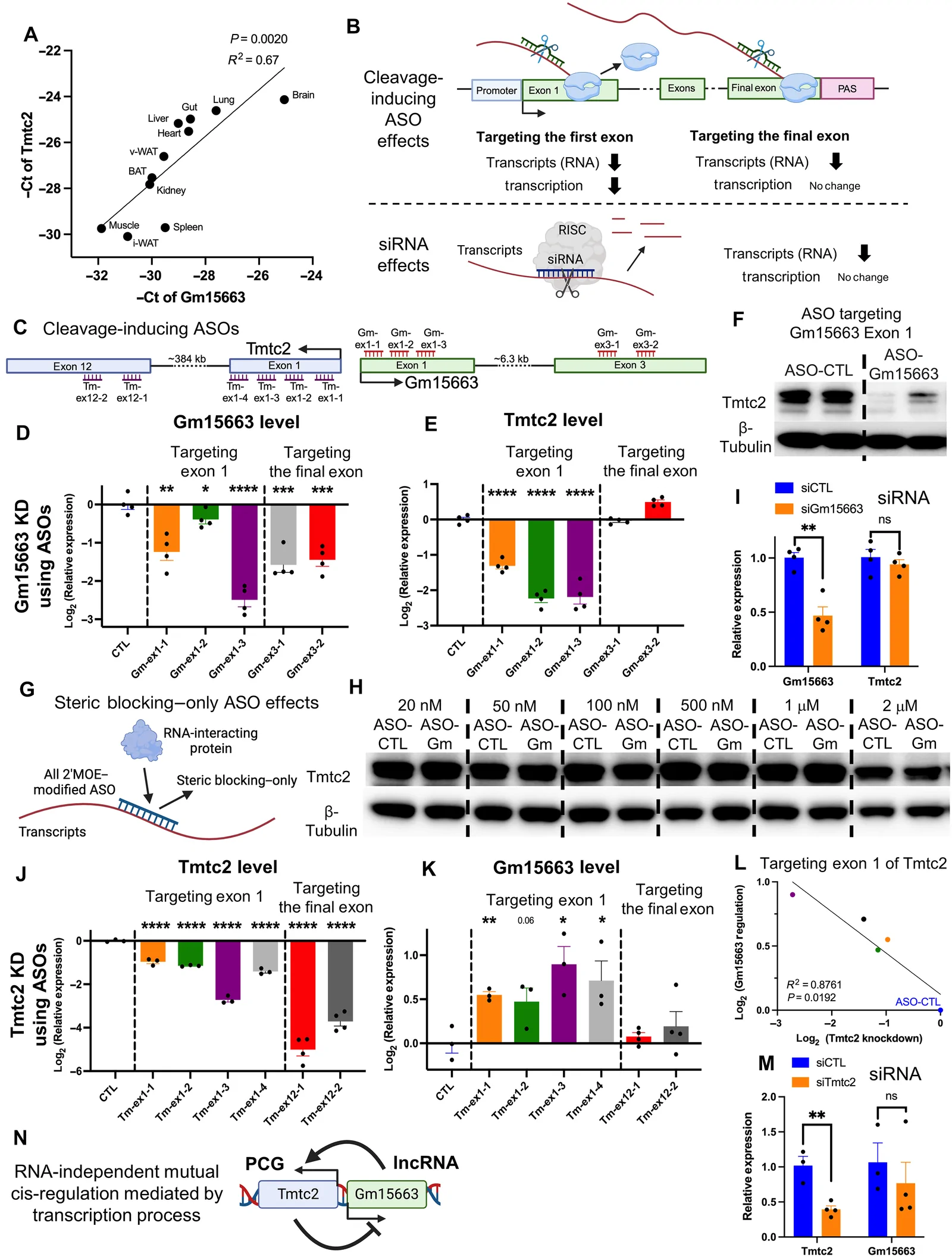
RNA-independent cis-autoregulatory circuits between *Gm15663* and *Tmtc2*. (**A**) Relative expression of *Gm15663*/*Tmtc2* across different tissues in mice. Expression was assessed by qRT-PCR, with the same amount of RNA for the RT reaction across all tissue samples. v-/i-WAT, visceral/inguinal white adipose tissue. BAT, brown adipose tissue. Linear regression analysis of the negative Ct values. (**B**) ASO treatment degrades mature transcripts and terminates transcription, while siRNA treatment only degrades mature transcripts. PAS, polyadenylation signal. Created in BioRender. X. Liu (2026) https://BioRender.com/3sygsij. (**C**) Positions of ASOs targeting *Gm15663*/*Tmtc2*. Gm-ex1/3, the first or the third (final) exon of *Gm15663*. Tm-ex1/12, the 1st or the 12th (final) exon of *Tmtc2*. (**D** and **E**) Relative expression of *Gm15663* (D) and *Tmtc2* (E) after treatment of primary hepatocytes with ASOs targeting different exons of *Gm15663*. (*n =* 4). (**F**) Protein level of *Tmtc2* in mouse primary hepatocytes treated with ASO targeting the first exon of *Gm15663*. Gapmer-ASOs were modified by LNA at both ends (see Materials and Methods). The targeting site of ASO-*Gm15663* is the same as Gm-ex1-3 in (C). β-Tubulin: loading control. (**G**) Steric blocking of RNA molecules by All-2′MOE–modified ASOs. Created in BioRender. X. Liu (2026) https://BioRender.com/3sygsij. (**H**) Protein levels of *Tmtc2* in mouse primary hepatocytes treated with All-2′MOE–modified ASO targeting *Gm15663*. β-Tubulin: loading control. (**I**) siRNA targeting *Gm15663* mature transcripts does not decrease *Tmtc2* expression in primary hepatocytes. (*n =* 4). (**J** and **K**) Relative expression of *Tmtc2* (J) and *Gm15663* (K) after treatment with primary hepatocytes with ASOs targeting the different exons of *Tmtc2*. (*n =* 3 to 4). (**L**) Correlation between *Tmtc2* knockdown and *Gm15663* up-regulation. Only ASOs targeting the first exon of *Tmtc2* and control are plotted. Raw data were the same as in (J) and (K). Linear regression analysis. (**M**) siRNA targeting *Tmtc2* transcripts does not increase *Gm15663* expression level. (*n =* 3 to 4). (**N**) Cis-regulation between *Gm15663* and *Tmtc2*.

Previous studies and our data above indicate that the expression of bidirectional lncRNAs and their neighboring PCGs are often correlated (*27*). Consistent with this, qRT-PCR of 11 different tissues from the mouse showed that the expression levels of *Gm15663* and *Tmtc2* were strongly positively correlated with each other across tissues, whereas there was no correlation with the expression level of the housekeeping gene 36B4 (Fig. 3A and fig. S6A).

To determine whether *Gm15663* cis-regulates *Tmtc2* and to elucidate the underlying mechanism, we used multiple approaches to manipulate the transcription level of *Gm15663* and assessed the impact on the transcription level of *Tmtc2*. First, we took advantage of the fact that cleavage-inducing antisense oligonucleotides (ASOs) can lower gene expression through two separate mechanisms: (i) knockdown of their mature target RNA transcripts and (ii) induction of the cleavage of nascent transcripts, which triggers the termination of the RNA Pol II transcription process and Pol II dissociation from the chromatin (Fig. 3B) (*61*, *62*). We treated primary hepatocytes with a panel of ASOs designed to target the first and the final (third) exons of *Gm15663* (Fig. 3C). As predicted, all five ASOs significantly reduced the levels of *Gm15663* (Fig. 3D). However, unexpectedly, only ASOs targeting the first exon of *Gm15663* (ASOs Gm-ex1-1, Gm-ex1-2, and Gm-ex1-3), but not those targeting the final exon (ASOs Gm-ex3-1 and Gm-ex3-2), produced significant down-regulation of *Tmtc2* (Fig. 3, E and F, and fig. S6B). It has been reported that ASOs targeting an early exon (exon 1) have strong effects on both knockdown of transcript levels and induction of transcription termination, while ASOs targeting the final exon can only induce the knockdown of transcript levels but have limited effects on transcription termination (Fig. 3B) (*63*). Precision Run-On (PRO)–qPCR showed that ASO Gm-ex1-3 was able to suppress the *Gm15663* transcription process (fig. S6C). Thus, these data suggest that while *Gm15663* expression cis-activates *Tmtc2* expression, it may be the *Gm15663* transcription process itself, rather than the resultant *Gm15663* transcripts, that contribute to the cis–up-regulation of *Tmtc2* transcription. Treatment of human hepatocytes with ASO targeting the first exon of *Gm15663*-human also reduced TMTC2 expression levels, indicating that this cis-regulation is conserved between mouse and human (fig. S6D).

To further confirm that it is the process of *Gm15663* transcription rather than the lncRNA molecule or its first exon RNA sequence, which cis–up-regulates *Tmtc2* transcription, we then only sterically block *Gm15663* transcripts at the first exon without disrupting its transcription process. To this end, we synthesized a steric blocking–only ASO targeting the same region of *Gm15663* as the ASO Gm-ex1-3. In this ASO, all of the nucleotides were modified with 2′-*O*-methoxyethyl group (All–2′-MOE–ASO) since All-2-MOE–ASOs target and only sterically block the transcripts but do not induce the cleavage of nascent or mature transcripts by ribonuclease H (*64*) and, thus, do not affect transcription process of the target gene (Fig. 3G). Although the original ASO Gm-ex1-3 targeting the first exon of *Gm15663* significantly reduced *Tmtc2* expression level (Fig. 3, D to F, and fig. S6B), the All-2-MOE–ASO with the same sequence did not reduce *Tmtc2* level across a wide range of tested concentrations (Fig. 3H), further indicating that *Gm15663* transcripts are dispensable for regulating *Tmtc2* level and that it is the transcription process itself that up-regulates *Tmtc2*. We further confirmed this using small interfering RNA (siRNA) (fig. S6E). In mammalian somatic cells, siRNAs can only promote the degradation of their target mature RNA but do not terminate the transcription process of their targets (Fig. 3B) (*65*, *66*). Consistent with the above data showing that cis–up-regulation of *Tmtc2* by *Gm15663* is not dependent on *Gm15663* transcripts, treatment of primary hepatocytes with siRNA against *Gm15663* lowered the level of *Gm15663* but did not decrease *Tmtc2* levels (Fig. 3I). Likewise, treatment of primary hepatocytes with plasmids that trans-overexpressed *Gm15663* transcripts by over 1000-fold from ectopic *Gm15663* loci did not change *Tmtc2* levels (fig. S6, F and G). Together, these data show that it is the *Gm15663* transcription process itself—but not the *Gm15663* transcripts—that maintains the expression level of *Tmtc2*.

To determine whether *Tmtc2* transcription process or its transcripts might reciprocally cis-regulate *Gm15663*, using similar knockdown approaches as above, we used ASOs targeting the first exon of *Tmtc2* to reduce *Tmtc2* transcripts and also to terminate the *Tmtc2* transcription process (Fig. 3C). In this case, ASO-mediated knockdown of *Tmtc2* significantly increased the level of *Gm15663*, with the levels of the increase in *Gm15663* showing an inverse correlation with the levels of Tmtc2 (Fig. 3, J to L). However, ASOs targeting the final (twelfth) exon of *Tmtc2* did not increase *Gm15663* levels (Fig. 3K). Together, the data indicate that *Tmtc2* cis-inhibits *Gm15663*, and this process may be dependent on *Tmtc2* transcription process, but not on the *Tmtc2* transcripts. To further confirm this concept, we treated primary hepatocytes with siRNA against *Tmtc2* to lower its transcript level without altering transcription process itself (fig. S6E). This treatment showed no effect on the level of *Gm15663* (Fig. 3M). Likewise, treatment of primary hepatocytes with plasmids that trans-overexpress *Tmtc2* transcripts did not alter *Gm15663* levels (fig. S6H). These data show that the cis-inhibition of *Gm15663* by *Tmtc2* relies on the *Tmtc2* transcription process but not on *Tmtc2* mature transcripts.

Together, these data demonstrate that *Gm15663* cis-promotes the expression of its bidirectional neighbor, *Tmtc2*, through its transcription process itself rather than its lncRNA transcripts. In a negative feedback loop, *Tmtc2* transcription, not its transcripts, cis-inhibits *Gm15663* expression. Collectively, these interactions create an elegant RNA-independent closed-loop autoregulatory circuit between the lncRNA and targeted PCG mediated by their transcription processes (Fig. 3N). Similar autoregulatory circuits are also observed in multiple other gene pairs, as will be described later.

### The first exon region of *Gm15663* functions as a promoter-proximal enhancer for *Tmtc2*, with its enhancer activity regulated by *Gm15663* transcription

We then ask how *Gm15663* transcription process itself maintains the expression level of *Tmtc2*. Compared to unidirectional promoters, bidirectional promoters have higher levels of enhancer chromatin signatures (*41*). Given these observations and our finding that it is the transcription process of lncRNA *Gm15663* that cis–up-regulates *Tmtc2*, we hypothesized that bidirectional lncRNA gene *Gm15663* may function as a promoter-proximal enhancer, with its activity and active chromatin environment being established by its own transcription.

**Fig. 4.**
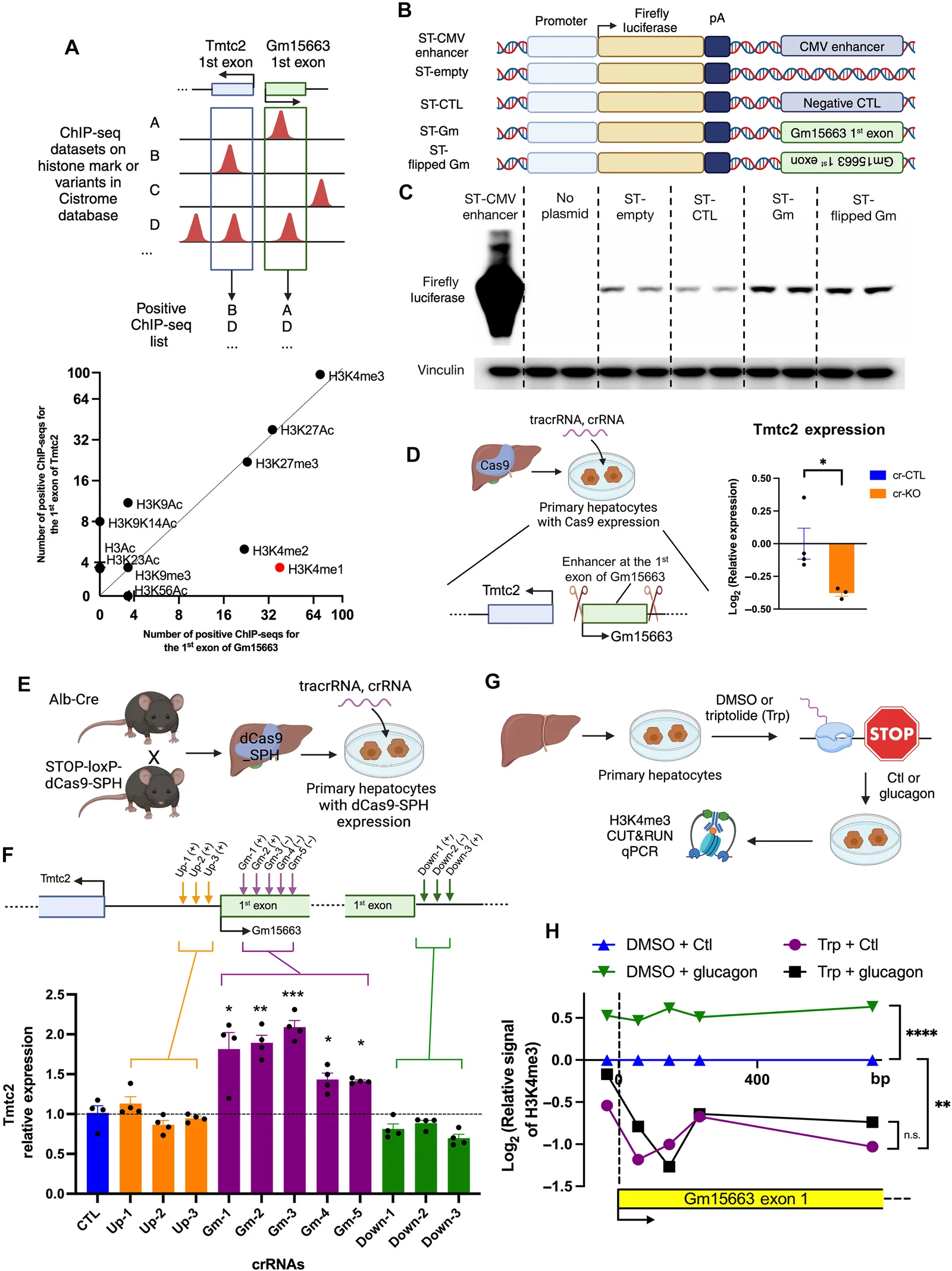
The first exon region of *Gm15663* functions as a promoter-proximal enhancer for *Tmtc2*, and the enhancer activity is regulated by *Gm15663* transcription. (**A**) The number of histone PTM ChIP-seq datasets showing peak signals on the first exons of *Gm15663* and *Tmtc2* in Cistrome database. Top: (A) to (D) schematically represent individual ChIP-seq datasets with or without peak signals at the first exon regions of *Tmtc2* and *Gm15663*; bottom: the image summarizes the aggregate number of positive ChIP-seq datasets for each histone modification at these two regions. Created in BioRender. X. Liu (2026) https://BioRender.com/3sygsij. (**B**) Schematic showing construction of plasmids for enhancer reporter assay in primary hepatocytes. pA, polyadenylation signals. ST-cytomegalovirus enhancer (Addgene 99312), ST-empty (Addgene 99297), ST-CTL (Addgene 99315). Created in BioRender. X. Liu (2026) https://BioRender.com/3sygsij. (**C**) Enhancer activity assay of the first exon of *Gm15663* in primary hepatocytes. The level of firefly luciferase was assessed by immunoblot. (**D**) Knockout of the first exon of *Gm15663* in primary hepatocytes decreases *Tmtc2* level. Expression was assessed by qRT-PCR. (*n =* 3 to 4). (**E**) Schematic showing construction of mice with hepatocyte-specific dCas9-SPH overexpression. Created in BioRender. X. Liu (2026) https://BioRender.com/3sygsij. (**F**) CRISPR-activation at the first exon of *Gm15663* in primary hepatocytes increases *Tmtc2* level. Expression was assessed by qRT-PCR. (*n =* 4). The orientations of the targeted sequences of crRNAs are labeled as +/− for plus/minus strand. (**G**) Schematic showing the measurement of H3K4me3 at the Exon 1 of *Gm15663* using CUT&RUN. Created in BioRender. X. Liu (2026) https://BioRender.com/3sygsij. (**H**) Levels of H3K4me3 at the exon 1 of *Gm15663* after glucagon or triptolide treatment. Significance was determined using a paired *t* test.

Active enhancers on mammalian chromatin are often associated with H3K4me1, p300, and H3K27Ac (*41*), the first two of which preferentially mark enhancers rather than promoters, and the last of which marks active regulatory sequences (*52*). Analysis of ChIP-seq datasets of H3K4me1, p300, and H3K27Ac on mouse liver (*67*–*69*) focusing on the *Gm15663* gene region indicated that the first exon region of *Gm15663* had high H3K4me1 and p300 levels, and a moderately high H3K27Ac level, compared to its neighboring region, i.e., the first exon of *Tmtc2* (fig. S7A). These suggest that this region has the signatures of an active enhancer. To more completely define the profile of histone PTMs around the *Gm15663* TSS region, we used the Cistrome Toolkit algorithm and Cistrome database (*57*, *58*) to identify ChIP-seq datasets of histone PTMs that exhibited binding signals in the first exon of *Gm15663* compared to its neighboring region (first exon of *Tmtc2*) (Fig. 4A). Despite these two regions being only 80 base pairs apart (fig. S3E), the number of H3K4me1 ChIP-seq datasets that detect binding signals in the first exon region of *Gm15663* was 18 times greater than the number of those detecting signals in the first exon region of *Tmtc2* (Fig. 4A, labeled red). As noted above, H3K4me1 is typically associated with enhancers, which suggests that the first exon of *Gm15663* may serve as a promoter-proximal enhancer for *Tmtc2*. To test this hypothesis, we constructed enhancer reporter plasmids in which the first exon of *Gm15663* (the first exon was defined in fig. S3, C to E), or its flipped version, was inserted downstream of the poly(A) signal of firefly luciferase (Fig. 4B). These reporter plasmids were transfected into primary hepatocytes, along with the strong cytomegalovirus enhancer as a positive control and a neutral DNA region as a negative control (*70*). The results showed that the first exon region of *Gm15663* inserted in either orientation enhanced the expression of firefly luciferase, indicating its role as an enhancer (Fig. 4C).

Given only 80 nucleotides separating the TSSs of *Gm15663* and *Tmtc2* (fig. S3E), it is possible that, in addition to serving as an enhancer, the first exon of *Gm15663* could also function as the core promoter of *Tmtc2*, initiating *Tmtc2* transcription. However, inserting the first exon of *Gm15663* in reverse orientation upstream of a promoter-less firefly luciferase in the plasmid, which simulates the native orientation of transcription between *Gm15663* and *Tmtc2* (fig. S7B), failed to initiate the transcription of firefly luciferase (fig. S7C), indicating that this region does not serve as the core promoter of *Tmtc2*.

To further define the enhancer function of the first exon region of *Gm15663* in the context of the local chromatin environment, we isolated primary hepatocytes from mice with liver-specific Cas9 overexpression (Fig. 2G) and transfected these cells with tracrRNA and crRNAs to knock out the first exon of *Gm15663* (Fig. 4D, S7D). Consistent with our hypothesis, knockout of the first exon region of *Gm15663* significantly reduced the expression of *Tmtc2* (Fig. 4D). To define the effect of activating *Gm15663*–first-exon enhancer region on *Tmtc2* expression, we also generated mice overexpressing dCas9-SunTag-p65-HSF1 (dCas9-SPH) specifically in the liver and then isolated primary hepatocytes from these mice and treated these cells with tracrRNA and crRNAs targeting the first exon of *Gm15663* to activate this region (Fig. 4E). Again, we found that the activation of *Gm15663*–first-exon enhancer by dCas9-SPH, but not regions that are upstream or downstream of the first exon, significantly up-regulated *Tmtc2* (Fig. 4F). Together, these data demonstrate that the first exon region of *Gm15663* functions as the promoter-proximal enhancer of *Tmtc2*, and targeting this enhancer can regulate *Tmtc2* expression level.

Enhancer transcription and enhancer H3K4me3 level are positively correlated with enhancer activity (*71*), and it has been reported that transcription determines local H3K4me3 level (*72*, *73*). Thus, to define whether the enhancer activity of the first exon of *Gm15663* is dynamically regulated in response to changes in *Gm15663* transcription, we first treated primary hepatocytes with glucagon, followed by CUT&RUN to assess the level of H3K4me3 at the *Gm15663*–first-exon enhancer (Fig. 4G). The data showed an increase of H3K4me3 levels upon glucagon treatment, indicating increased enhancer activity associated with up-regulation of *Gm15663* (Fig. 4H). To further explore the role of *Gm15663* transcription in this regulation, we inhibited transcription with triptolide followed by glucagon treatment and CUT&RUN analysis on H3K4me3 (Fig. 4G). The data showed that triptolide decreased H3K4me3 at the *Gm15663*–first-exon enhancer, and its level was not significantly increased by glucagon when *Gm15663* transcription was not up-regulated (Fig. 4H). These data confirm that the dynamic regulation of *Gm15663* transcription is crucial for controlling the enhancer activity. In addition, we also analyzed H3K4me3 ChIP-seq data on liver samples taken at different ZT times in the circadian cycle (*68*), i.e., as the level of *Gm15663* transcription oscillated (fig. S2E). The data showed that the levels of H3K4me3 at the *Gm15663*–first-exon enhancer were lower during the light-off period in the circadian cycle when *Gm15663* transcription level was lower, compared to the light-on period (fig. S7E). This is consistent with the hypothesis that this enhancer activity is dynamically regulated following the *Gm15663* transcriptional changes.

### Internal reciprocal cis-regulation within bidirectional gene pair controls hepatic lipid metabolism

**Fig. 5.**
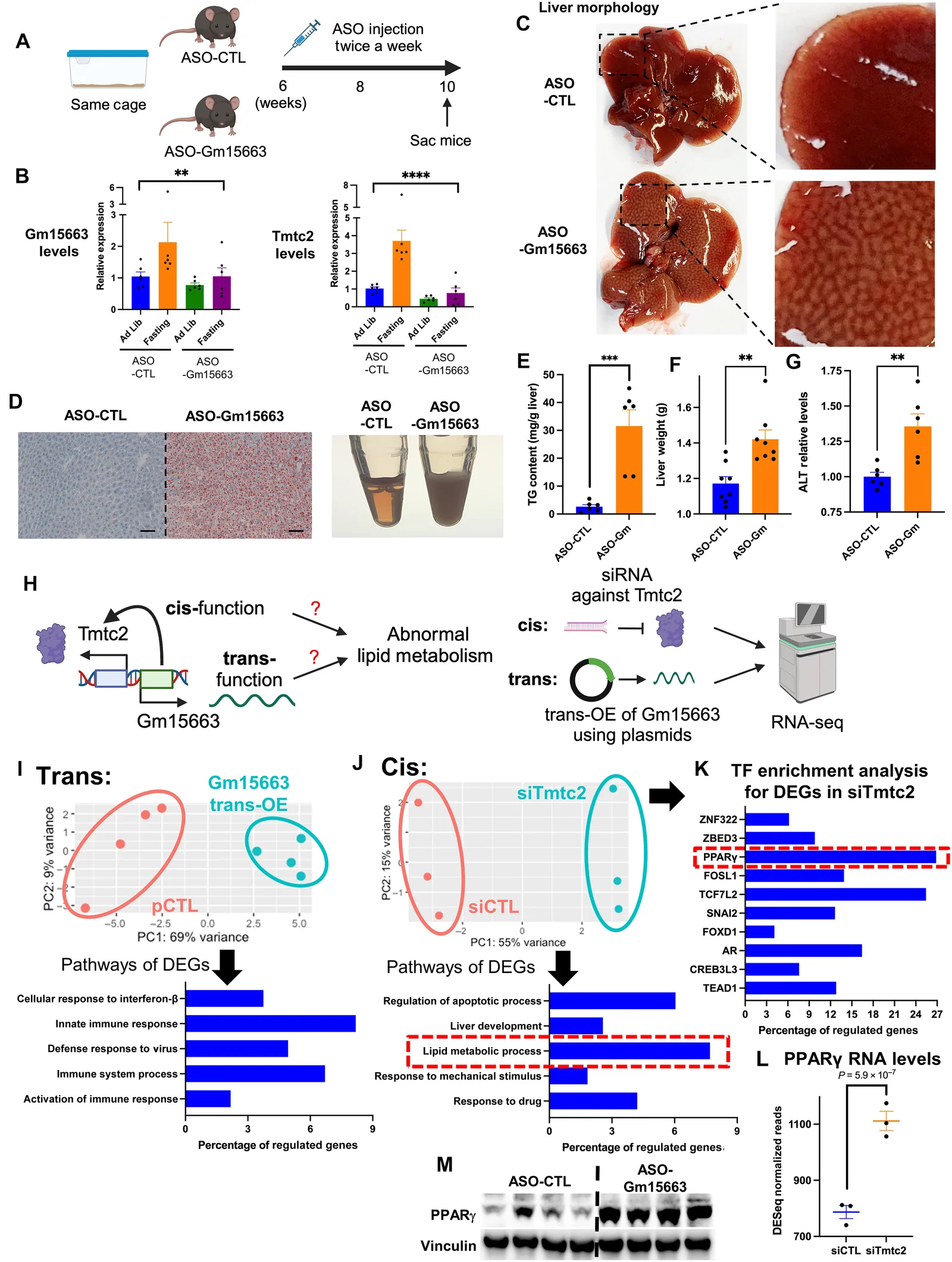
*Gm15663* controls lipid metabolism in liver. (**A**) Schematic of in vivo ASO treatment in male C57BL/6J mice on chow diet. Created in BioRender. X. Liu (2026) https://BioRender.com/14xftdd. (**B**) Relative expression levels of *Tmtc2* and *Gm15663* in livers of mice treated with ASO-CTL or ASO-*Gm15663* in vivo under ad libitum or after overnight fasting. (*n =* 6). Significance was determined using one-way analysis of variance (ANOVA). (**C** and **D**) Liver morphology (C), hepatic Oil Red O staining, and supernatants of liver homogenate (D). Scale bar, 20 μm. (**E**) Hepatic triglyceride (TG) levels. (*n =* 6). (**F** and **G**) Liver weight (F) and circulating ALT levels (G) in mice treated with ASOs. [(F) *n =* 8, (G) *n =* 6]. Gm, *Gm15663*. (**H**) Schematic showing identification of cis/trans functions of *Gm15663* using transcriptomic analysis. OE, overexpression. Created in BioRender. X. Liu (2026) https://BioRender.com/14xftdd. (**I**) Principal components analysis of transcriptomics from AML12 hepatocytes transfected with control pcDNA plasmids (pCTL) or pcDNA plasmids overexpressing *Gm15663* (trans-OE) (*n =* 4). (**J**) Principal components analysis of transcriptomics from primary hepatocytes transfected with negative control siRNA (siCTL) or siRNA targeting *Tmtc2* (si*Tmtc2*, targeted positions shown in fig. S6E) (*n =* 3). For (I) and (J), DEGs were identified with a threshold of *P* < 0.05 and a fold change greater than 1.25 for up- or down-regulation. Pathways enriched in DEGs were identified using DAVID GO analysis (https://davidbioinformatics.nih.gov/), with the top 5 pathways ranked by *P* value displayed. (**K**) TF enrichment analysis of DEGs in si*Tmtc2* versus siCTL using chEA3. (**L**) Pparg mRNA levels in si*Tmtc2* and siCTL. Significance and *P* values were determined using DESeq2. (**M**) Protein levels of PPARγ in livers of mice treated with ASO-*Gm15663* and ASO-CTL. Vinculin, loading control.

To determine the physiological role of the internal cis-regulation of *Gm15663*/*Tmtc2* in hepatic metabolism in vivo, we initially treated mice with Gapmer ASOs targeting the first exon of *Gm15663* to block its transcription in liver in vivo for 4 weeks (Fig. 5A). Consistent with the in vitro data, *Gm15663* deficiency disrupts the regulation of both *Gm15663* and *Tmtc2* during fasting in liver (Fig. 5B), and this also resulted in a significant reduction of both *Gm15663* and *Tmtc2* expression levels (Fig. 5B). Knockdown of *Gm15663* resulted in significantly increased hepatic lipid accumulation (Fig. 5, C to E), an ∼20% increase in liver weight (Fig. 5F), and an increase in the circulating level of alanine transaminase (ALT) indicating liver injury (Fig. 5G). The hepatic steatosis was also accompanied by decreased body weight gain, reduced white adipose tissue mass (fig. S8, A to E), despite unchanged levels of circulating free fatty acid and triglyceride (fig. S8, F and G), indicating that *Gm15663* is important in the maintenance of both hepatic and systemic metabolic homeostasis.

To determine whether the effects of *Gm15663* deficiency on hepatic lipid accumulation are contributed by the cis or trans functions of *Gm15663*, we knocked down *Tmtc2* directly using siRNA (cis effects) or overexpressed *Gm15663* transcripts (trans effects) in hepatocytes in vitro followed by RNA sequencing (RNA-seq) analysis (Fig. 5H). The differentially expressed genes (DEGs) were then identified and subjected to GO pathway analysis (fig. S8, H and I). Our data showed that the top pathways associated with *Gm15663* trans function primarily involve immune response and virus defense (Fig. 5I), whereas the pathway that contains the most regulated genes of si*Tmtc2* (cis function of *Gm15663*) was “lipid metabolic process” (Fig. 5J). Furthermore, TF enrichment analysis of the DEGs in si*Tmtc2* identified peroxisome proliferator–activated receptor γ (PPARγ), a critical TF-driving lipid accumulation, as a key regulator controlling the highest portion of DEGs by *Tmtc2* knockdown (Fig. 5K). PPARγ was significantly up-regulated in the RNA-seq data from *Tmtc2* knockdown, reinforcing its role in contributing to the observed DEGs (Fig. 5L). Consistent with this, in the livers of ASO-*Gm15663*–treated mice, significantly higher protein and mRNA levels of PPARγ were also validated (Fig. 5M and fig. S9). To further characterize how *Gm15663* inhibition affects hepatic lipid metabolism, we measured the expression of genes involved in lipid metabolism in livers from ASO-treated mice. ASO-*Gm15663* treatment increased the expression of multiple genes involved in lipid uptake and triglyceride synthesis (fig. S9). ASO-*Gm15663* also increased the expression of many critical genes involved in de novo lipogenesis and cholesterol synthesis (fig. S9).

Together, these data suggest that the abnormal hepatic lipid metabolism observed in the ASO-*Gm15663*–treated mice is driven by the cis function of *Gm15663* in regulating *Tmtc2* rather than by the trans function of *Gm15663*, indicating the physiological importance of cis-regulatory circuits within bidirectional gene pairs in hepatic metabolism in vivo.

### Reciprocal cis-autoregulatory circuits across metabolically regulated lncRNA/PCG and PCG/PCG pairs

**Fig. 6.**
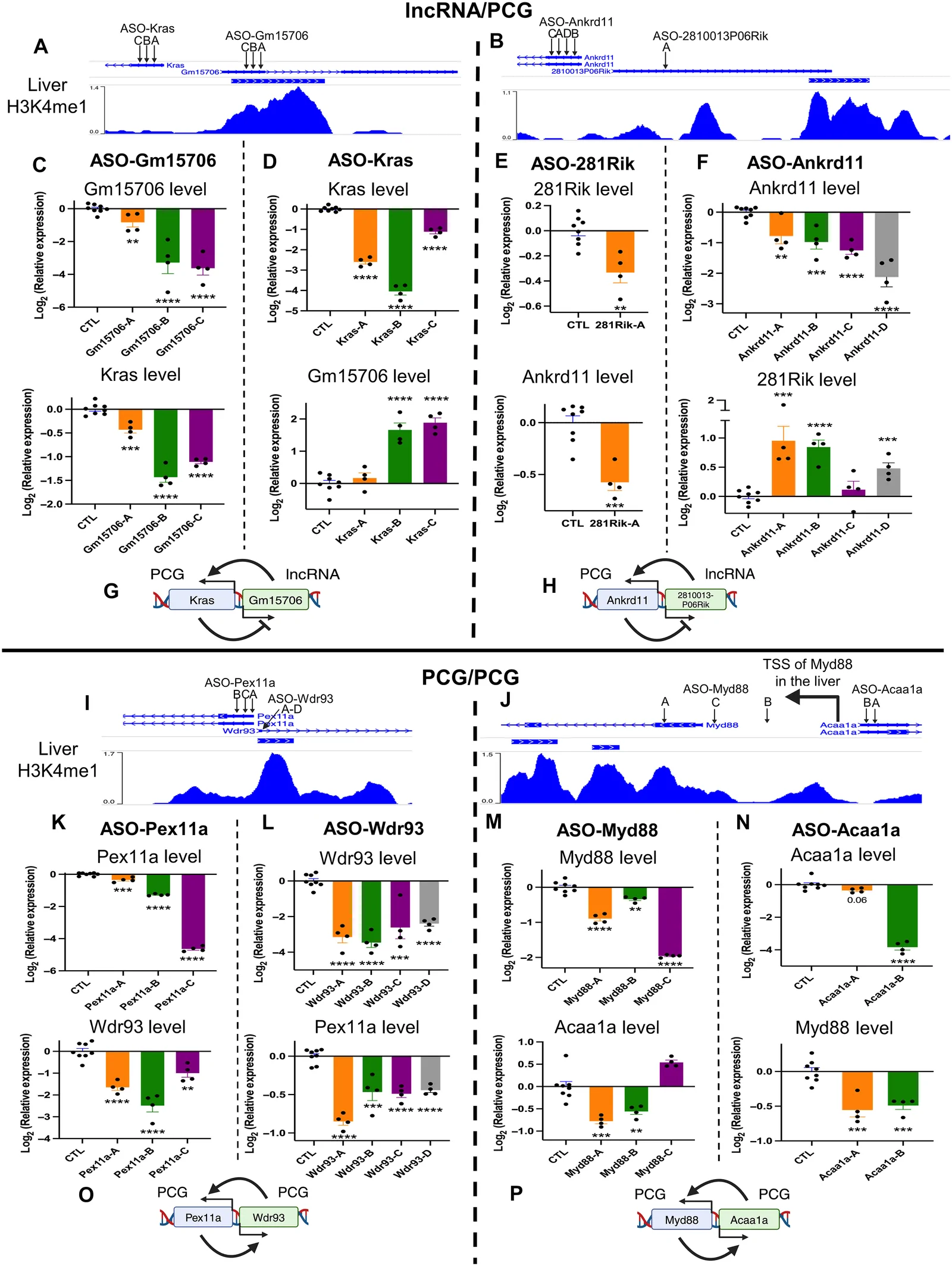
Mutual cis-regulation in other lncRNA/PCG and PCG/PCG pairs. (**A** and **B**) H3K4me1 ChIP-seq data in the regions close to the shared *Gm15706*/*Kras* (A) or *2810013P06Rik*/*Ankrd11* (B) promoters in the liver. See Materials and Methods for the origins of ChIP-seq datasets. Positions of different ASOs targeting the first exons of *Gm15706* and *Kras* (A) or *2810013P06Rik* and *Ankrd11* (B). (**C** and **D**) Relative expression levels of *Gm15706* and *Kras* after treatment of primary hepatocytes with ASOs targeting the first exon of *Gm15706* (C) or *Kras* (D). (*n =* 4 or 8). (**E** and **F**) Relative expression levels of *2810013P06Rik* and *Ankrd11* after treatment of primary hepatocytes with ASOs targeting the first exon of *2810013P06Rik* (E) or *Ankrd11* (F). (*n =* 4 or 8). (**G** and **H**) Schematic showing that the transcription process of bidirectional lncRNA cis–up-regulates its neighboring PCG, whereas the transcription process of the PCG cis-inhibits the lncRNA. Created in BioRender. X. Liu (2026) https://BioRender.com/14xftdd. (**I** and **J**) H3K4me1 ChIP-seq data in regions close to shared *Pex11a*/*Wdr93* (I) and *Myd88*/*Acaa1a* (J) promoters in the liver. See Materials and Methods for the origins of ChIP-seq datasets. Positions of different ASOs targeting the first exons of *Pex11a*/*Wdr93* (I) and *Myd88*/*Acaa1a* (J). The TSS of *Myd88* in the liver was determined by GRO-seq (GSM1437738). (**K** and **L**) Relative expression levels of *Pex11a* and *Wdr93* after treatment of primary hepatocytes with ASOs targeting the first exon of *Pex11a* (K) or *Wdr93* (L). (*n =* 4 or 8). (**M** and **N**) Relative expression levels of *Myd88* and *Acaa1a* after treatment of primary hepatocytes with ASOs targeting the first exon of *Myd88* (M) or *Acaa1a* (N). (*n =* 4 or 8). (**O** and **P**) Schematic showing that in a bidirectional PCG/PCG pair, the transcription process of one PCG cis–up-regulates the other PCG. Created in BioRender. X. Liu (2026) https://BioRender.com/14xftdd. Expression was assessed by qRT-PCR.

As noted above, metabolic transitions regulate multiple lncRNA/PCG gene pairs, as well as multiple PCG/PCG pairs. Thus, we asked whether this mutual cis-autoregulatory circuits between *Gm15663* and *Tmtc2* can be observed in other metabolically regulated bidirectional gene pairs. Similar to the *Gm15663*/*Tmtc2* pair, high levels of H3K4me1 were identified in close proximity downstream of the lncRNA TSS in other bidirectional lncRNA/PCG pairs coordinately regulated in metabolic states in the liver, including the *Gm15706*/*Kras* and *2810013P06Rik*/*Ankrd11* (Fig. 6, A and B), supporting the notion that a bidirectional lncRNA gene may serve as the promoter-proximal enhancer for the neighboring PCG. To investigate whether mutual cis-regulation of the lncRNA and PCG also exists in these bidirectional gene pairs, we attenuated the transcription of individual genes in each pair by treating mouse primary hepatocytes with ASOs designed to target the promoter-proximal regions, i.e., the first exons, which are shortly downstream of the TSSs (Fig. 6, A and B). The data revealed that for these bidirectional lncRNA/PCG pairs, knocking down the transcription of lncRNA decreased the transcriptional level of the neighboring PCG (Fig. 6, C and E), whereas knocking down the transcription of PCG increased the transcriptional level of the lncRNA (Fig. 6, D and F), mirroring what was observed for *Gm15663*/*Tmtc2* (Figs. 6, G and H, and 3N) and indicating that this mechanism extends beyond a single locus. As noted above, in addition to lncRNA/PCG pairs, coordinated regulation and promoter-proximal enhancer signatures were also identified in the bidirectional PCG/PCG pairs regulated by metabolic states, such as *Pex11a*/*Wdr93* and *Myd88*/*Acaa1a* (Fig. 6, I and J). Again, using ASOs targeting promoter-proximal regions of these genes to block their transcription process, we demonstrated that mutual cis-regulation also exists for two genes in PCG/PCG pairs (Fig. 6, K to N), but, in this case, regulation in both directions was positive (Fig. 6, O and P).

**Fig. 7.**
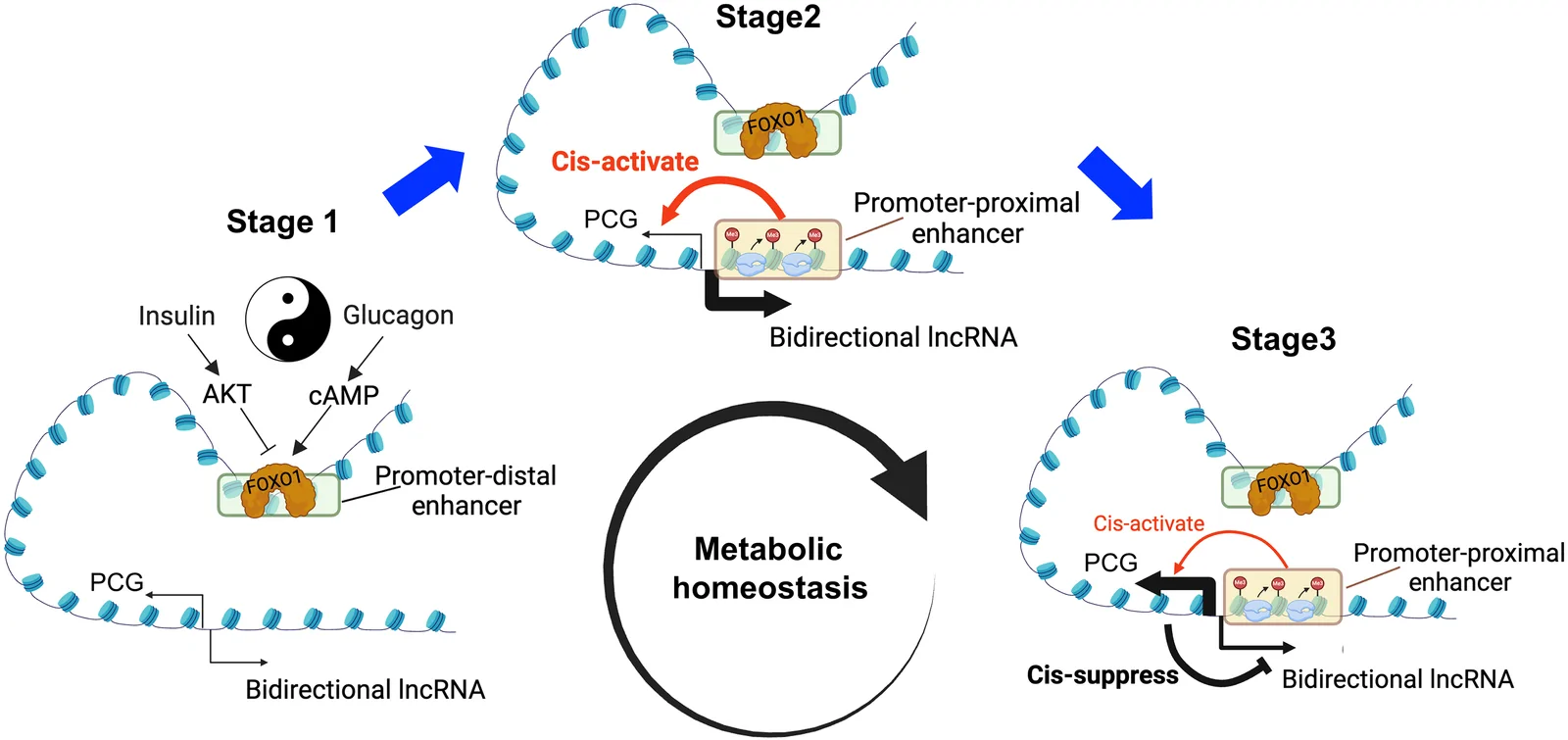
RNA-independent cis-autoregulatory circuits between lncRNA and PCG maintain metabolic homeostasis. Schematic depicting three stages in the RNA-independent regulatory circuits between lncRNA and PCG. Stage 1: FOXO1 binds to promoter-distal enhancers and the chromatin topological looping between the enhancers and promoter forms. Stage 2: The transcription process of bidirectional lncRNA increases the activity of the promoter proximal enhancer, which in turn cis-up-regulates the PCG. Stage 3: The transcription process of PCG cis-suppresses bidirectional lncRNA. Created in BioRender. X. Liu (2026) https://BioRender.com/14xftdd.

Together, these data demonstrate that mutual cis-regulation is a mechanism shared by multiple metabolically regulated lncRNA/PCG, and even PCG/PCG pairs. This regulatory mechanism is dependent on the transcription process and mediated via an associated promoter-proximal enhancer, but this is independent of the RNA transcripts or proteins. This mechanism creates intricate closed-loop reciprocal autoregulatory circuits, which integrates mutual cis-regulation within these pairs and coordinated hormonal regulation mediated by 3D genome architecture, to achieve the precise control in dynamic hormonal regulation in metabolic homeostasis (Fig. 7).

## DISCUSSION

Metabolic homeostasis is a complex and highly integrated process involving insulin, glucagon, and multiple other hormones. A critical element of this regulation is the control of gene expression. Many conserved lncRNAs and PCGs involved in metabolic regulation and other aspects of cellular and organismal homeostasis are closely juxtaposed with PCGs, forming bidirectional gene pairs. In this study, by focusing on a conserved lncRNA, *Gm15663*, we showed that bidirectional lncRNA controls hepatic metabolism by cis-regulating its neighboring PCG. Mechanistically, this process involves RNA-independent cis-autoregulatory circuits between *Gm15663* and *Tmtc2*, mediated by transcription process itself and the activity of associated promoter-proximal enhancer. Similar mutual cis-regulatory circuits dependent on transcription process are also observed in many other lncRNA/PCG and PCG/PCG pairs, underscoring their importance in maintaining metabolic homeostasis.

Our data provide new insight into the molecular mechanism of gene regulation by insulin and glucagon in the maintenance of metabolic homeostasis. It has been shown FOXO1 functions as a key TF that integrates the antagonistic actions on gene regulation by insulin and glucagon, as well as the abnormal gene expression profile observed in insulin resistant states, such as T2D (*2*, *5*, *10*). However, how FOXO1 has such broad effects remains unclear since the promoters of many FOXO1-regulated genes do not contain FOXO1-binding motifs. Our work shows that, in addition to binding to core promoters, FOXO1 is also able to bind to promoter-distal enhancer regions that form dynamic chromatin topological loops between the promoter and FOXO1-binding enhancers to regulate gene transcription. Thus, our study adds an important new layer of the FOXO1 regulatory mechanisms, similar to what has been demonstrated for other TFs, including YY1, ERα, and REV-ERBα (*7*, *74*, *75*). For the *Gm15663*/*Tmtc2* gene pair, these FOXO1-binding enhancers are liver specific, but further studies will be needed to determine whether FOXO1 can function as a pioneer factor in the liver to establish a liver-specific enhancer profile to achieve liver-specific gene regulation by metabolic hormones. It will also be important to further identify whether other metabolic-responsive TFs regulate gene expression through similar reorganization of genome topology and its associated interaction between distal elements.

Our data also demonstrate a less-studied mechanism, observed across multiple tested bidirectional gene pairs by which an lncRNA can regulate its neighboring PCG. This is dependent on lncRNA transcription process, which helps establish active chromatin environment at the enhancer located in the lncRNA region and regulates the enhancer activity. Multiple mechanisms have been proposed on how a bidirectional lncRNA regulates its neighbor, and they are not mutually exclusive (*76*–*79*). While effects of the lncRNA transcripts have received much attention, the role of lncRNA transcription process itself in regulating the neighboring PCG has been much less studied. It has been reported that the 5′ splice site of bidirectional lncRNAs can play a role in cis-activating its neighbor (*28*, *80*), while the precise RNA sequence beyond the initial splice signal is not required. It has also been reported that the DNA region containing a bidirectional lncRNA may also contain super-enhancer signature (*41*), and the transcription process of a bidirectional lncRNA is important in the elongation of the RNA Pol II at the enhancer locus, thus maintaining the neighboring gene transcription (*27*). Our study supports this model in a generalizable way, in the context of metabolically regulated gene pairs. We also show that the cis-regulation can also be expanded to bidirectional PCGs since this mechanism is only dependent on the transcription process and its associated enhancer signatures, which does not involve specific RNA functional sequences. This mechanism also helps to explain why bidirectional gene pairs exhibit higher transcription burst frequency compared to unidirectional promoters since burst frequency is primarily encoded in enhancers (*81*, *82*).

In addition to the transcription of the lncRNA regulating the neighboring PCG, we show that the transcription process of the PCG is also able to cis-inhibit the bidirectional lncRNA as an internal negative feedback, creating a closed-loop reciprocal autoregulatory circuit. This cis-inhibition on the lncRNA transcription by PCG may occur through several potential mechanisms. One is that the transcription of the lncRNA and PCG may share a common pool of the transcriptional “resources” at the common promoter, such as Pol II, mediator, and general TFs. When the transcription of PCG is at a high level, it may consume these factors, thereby reducing the pool and inhibiting lncRNA transcription (*83*). Another potential mechanism is that an excess of transcribed RNA in the transcriptional condensate could result in the dissolution of the condensate, leading to a reduction of local transcription (*76*).

While we have demonstrated transcription-mediated cis-autoregulatory circuits for three lncRNA/PCG pairs and two PCG/PCG pairs, a systematic, genome-wide perturbation analysis would be valuable for further establishing the prevalence of this regulatory mechanism for all bidirectional gene pairs. However, such an approach is currently not feasible in principle in this context. Unlike conventional genome-wide perturbations that rely on a uniform phenotypic or molecular readout, interrogation of bidirectional gene pairs requires pair-specific readouts, as perturbation of one gene must be evaluated through the transcriptional response of its paired genomic neighbor. This cannot currently be achieved using standardized screening strategies, such as CRISPR- or siRNA-screen. In addition, a comprehensive genome-wide analysis for transcription-mediated gene regulation would necessitate the design, optimization, and validation of highly efficient perturbation reagents targeting the transcription per se for thousands of genes in a cell type–specific context, posing substantial technical challenges. Nonetheless, developing an approach for genome-wide perturbation of bidirectional gene pairs should be an important future goal for the field.

We note that while this transcription-dependent cis-regulation was mechanistically dissected using multiple orthogonal methods primarily in isolated hepatocytes and supported by more physiological in vivo ASO experiments, future in vivo studies using ASOs targeting final exons or genetic approaches that selectively distinguish mature RNA depletion from transcriptional disruption will be valuable for further corroborating this mechanism in the intact animal. The cis-regulatory mechanism by lncRNAs identified here may also offer novel insights into the explanation of unique patterns observed in lncRNA gene evolution. Most lncRNA gene sequences have evolved rapidly, and the conserved region is short and biased to 5′ end (*84*). For bidirectional gene pairs, our data show that it is the lncRNA transcription process that regulates a 5′-proximal enhancer that is crucial for the neighboring PCG transcription, and this is independent of the transcription of later exons or mature RNA transcripts. This would help explain the 5′-biased conservation of lncRNA gene evolution. This mechanism also uncouples the lncRNA gene functions with the stability of RNA transcripts and their distribution, indicating that the cis and trans functions of an lncRNA gene can be highly distinct. Thus, when identifying disease-associated noncoding genes, it is important not only to simply focus on defining disease-associated mature transcripts using methods such as RNA-seq but also to assess the disease-associated RNA transcription process using methods detecting nascent transcripts, such as GRO/PRO-seq and transient transcriptome sequencing. In addition, our data suggest that when the transcription process of an lncRNA gene contributes to disease-related gene regulation, approaches that modulate transcription process itself or the associated local chromatin environment may be worth considering. These could include ASOs targeting promoter-proximal regions or CRISPR-based inhibition or activation strategies. Further studies will also be needed to evaluate their therapeutic potential in disease settings.

Notably, genes trans-regulated by *Gm15663* are distinct from its cis-regulated genes. These trans-regulated genes are predominantly immune related, primarily in interferon-response pathways. Given the reported interplay between interferon signaling and liver metabolism (*85*, *86*), the trans functions of *Gm15663* may also contribute to liver metabolic physiology. Thus, while our study primarily defines an RNA-independent cis-regulatory function of *Gm15663* at the bidirectional promoter locus, the potential trans functions of *Gm15663* on the interferon pathways and their associated metabolic effects warrant further investigation.

In summary, this study identifies a previously unappreciated aspect in the regulatory mechanisms within bidirectional lncRNA/PCG and PCG/PCG pairs, namely the cis-autoregulatory circuits dependent on gene transcription process rather than transcripts. Our findings also prompt a revision of the canonical concept regarding the role of lncRNAs and PCGs in metabolism, highlighting transcription-mediated circuits as important regulators. Together with external factors, such as hormone-TF–mediated chromatin topological alterations, these mechanisms form an integrated, closed-loop control circuit that allows more precise and coordinated transcriptional regulation of lncRNAs and PCGs, allowing for tight regulation of metabolic homeostasis.

## MATERIALS AND METHODS

### Animals

All mice were housed in a room with controlled climate conditions, and a 12-hour light/dark cycle with lights turned on at 7:00 a.m. Mice were provided with a standard chow diet unless otherwise specified. The sex and age of mice are specified in figure legends. All studies were conducted using protocols approved by the Joslin Diabetes Center Animal Welfare Committee.

### Primary hepatocyte isolation

Mouse livers were perfused with Krebs-Ringer buffer containing 100 μM EGTA (Sigma-Aldrich) followed by the perfusion with 60 mg of type I collagenase (Thermo Fisher Scientific) dissolved in 50 ml of Dulbecco’s modified Eagle’s medium (DMEM)/F12 medium. The digested liver was then gently disrupted in a sterile petri dish using fine-tipped forceps to release single cells followed by filtering through a 100-μm strainer. Isolated hepatocytes were precipitated at 50*g* for 3 min and were resuspended in 10 ml of DMEM. Ten milliliters of Percoll solution [9 ml of Percoll +1 ml of 10× phosphate-buffered saline (PBS)] was added, and healthy hepatocytes were precipitated by centrifugation at 700*g* for 10 min. The isolated hepatocytes were then washed by PBS twice and seeded at the density of 2.5 to 3.5 × 10^5^/ml in DMEM containing 20% fetal bovine serum (FBS) onto collagen-coated plates. After 4 hours, floating cells were removed, and the medium was changed to Lonza HCM hepatocyte culture medium (CC-3198) or kept in DMEM with 20% FBS.

### RNA isolation and qPCR

RNA was isolated using TRIzol or RNeasy Mini Kit (Qiagen). cDNA synthesis was performed using a High-Capacity cDNA Reverse Transcription Kit (Thermo Fisher Scientific), following the instructions of these kits from manufacturers. For cDNA synthesis, 0.5 to 1.5 μg of RNA and random hexamer primers were used in each reaction. qPCR was performed using iQ SybrGreen Supermix (Bio-Rad). Two technical replicates were used for each reaction. Each 10-μl qPCR reaction contained forward (F) and reverse (R) primers at 0.25 μM, 5 μl of iQ SybrGreen Supermix and 5 μl of diluted cDNA. Primers for qPCR of mouse genes were as follows: *Gm15663* (F: GACGCGGAGAGGTAGAACAG, R: GGCCCTTCCATCCCAATTCA), *Tmtc2* (F: CCTTGTATCTCAACACCCTGAGT, R: AGTCCCCCAAAAGTCATTGTAGA), *G6pc* (F: CGACTCGCTATCTCCAAGTGA, R: GTTGAACCAGTCTCCGACCA), 36B4 *(Rplp0)* (F: AGATTCGGGATATGCTGTTGGC, R: TCGGGTCCTAGACCAGTGTTC), *Gm15706* (F: GACGCCAGATGAAGGAAGAA, R: GCTCAAGGCAGACTGAGATAC), *Kras* (F: CAAGAGCGCCTTGACGATACA, R: CCAAGAGACAGGTTTCTCCATC), *2810013P06Rik* (F: GAGGTGACCTTTCTTCCCTATTT, R: AGGACAGCTACTCATCACAAAG), *Ankrd11* (F: AGTCTACAGTGTGTCAGAAGGG, R: TGCAAAGTCCTTGACGTTCAC), *Pex11a* (F: GACGCCTTCATCCGAGTCG, R: CGGCCTCTTTGTCAGCTTTAGA), *Wdr93* (F: TGTTCCTATCGACGAAATGGGT, R: CTGCTTGCTGCTATCATCCAC), *Myd88* (F: TCATGTTCTCCATACCCTTGGT, R: AAACTGCGAGTGGGGTCAG), and *Acaa1a* (F: TCTCCAGGACGTGAGGCTAAA, R: CGCTCAGAAATTGGGCGATG). Primers for qPCR of human genes were as follows: *Gm15663*-Human (F: GAAAGGAGGTCGAGGAGGA, R: CAGCTCTTGGCACACACA); *TMTC2* (F: CATGGCTGGCTTGATGTTTG, R: GCAAGGAGAGGAGAAAGAAGAG); and *RPLP0* (F: GAAACTCTGCATTCTCGCTTCC, R: GACTCGTTTGTACCCGTTGATG). Primers were synthesized by Integrated DNA Technologies.

### ASO synthesis and transfection

ASOs were synthesized by Integrated DNA Technologies. All ASOs were fully phosphorothioated. A nontargeting ASO was used as the negative control. ASO-CTL: GCGACTATACGCGCAATATG. ASOs against *Gm15663* were as follows: Gm-ex1-1 (AGTACCAGGCTGGAGCAGCT), Gm-ex1-2 (TGAAGAAGAGAGTGGGGCCA), Gm-ex1-3 (GTAGAGGGTTGCATTAGCGT), Gm-ex3-1 (GCTGGGTTACTTTGTCTGGC), and Gm-ex3-2 (GCAAGACAGTTTTCTCAGCC). ASOs against *Tmtc2* were as follows: Tm-ex1-1 (CTGCGTGGCCAGCCCACCAC), Tm-ex1-2 (GAGGATCTGGGACCAGCGGG), Tm-ex1-3 (ACCGCCTTCCCTCCGGCTTG), Tm-ex1-4 (CAGCACTCAGGGTGTTGAGA), Tm-ex12-1 (GCGGAGATTAGACTGA), and Tm-ex12-2 (CCTCTACGAGTGGTTA). For Tm-ex12-1/2 and experiments in fig. S6 (B to D) and Fig. 3F, Gapmer ASOs were used. The Gapmer ASOs were composed of 16 nucleotides, with the first three and the last three nucleotides modified using locked nucleic acid (LNA) base. LNA-ASO-CTL: GACTATACGCGCAATA, LNA-ASO-*Gm15663*: AGAGGGTTGCATTAGC. The steric blocking–only ASO against *Gm15663* was fully modified with 2’-MOE with the same sequence as Gm-ex1-3. LNA modified Gapmer ASOs were used to target *Gm15706*/*Kras*, *2810013P06Rik*/*Ankrd11*, *Pex11a*/*Wdr93*, and *Myd88*/*Acaa1a*. The sequences of these ASOs were: ASO-negative-CTL (GGCTACTACGCCGTCA), *Gm15706*-A (CACTTCCTACAAAGCG), *Gm15706*-B (TCCATGTGGTATCCGG), *Gm15706*-C (GTCTACACAGAGAGAC), *Kras*-A (GGGTCCGAAATGGCGC), *Kras*-B (CTCCGGGTCCGAAATG), *Kras*-C (TCGCTCCGGGTCCGAA), *2810013P06Rik*-A (ACGATGCGACTTTGCT), *Ankrd11*-A (TCGGTCCATCGCGCTC), *Ankrd11*-B (CTGGCTCCAGGACCCA), *Ankrd11*-C (TCGGCGTGCAGGAGCT), *Ankrd11*-D (ATCGCGCTCTGTCGCG), *Pex11a*-A (TCGGAAACGCGGCTTT), *Pex11a*-B (GCGGGAACAGATTAGT), *Pex11a*-C (GTGGGACTTCGTCTCT), *Wdr93*-A (TTGTGGCTTGGCGTCG), *Wdr93*-B (TCTTGTGGCTTGGCGT), *Wdr93*-C (TCTTGTGGCTTGGCGT), *Wdr93*-D (AAGTCTTGTGGCTTGG), *Myd88*-A (CTCACGGTCTAACAAG), *Myd88*-B (GGAAGTAGTAACCCGA), *Myd88*-C (GAATCTAGACTACGGG), *Acaa1a*-A (AGCCTTCTACGTCGCA), and *Acaa1a*-B (CACTCCTAGGTACAAC). LNA-modified Gapmer ASOs were used to target *Gm15663*-Human: ACCGGACTTACGCCTG.

ASOs were transfected to cells using Lipofectamine RNAiMAX (Thermo Fisher Scientific). Briefly, cells were seeded to achieve 80% confluence at the time of transfection. For each well in a 12-well plate, 1 μM ASO (or 100 nM LNA-ASO) were transfected with 5 μl of Lipofectamine RNAiMAX. For 6- or 24-well plates, the amount of ASO was adjusted proportionately.

### *Gm15663* ASO in vivo treatment

C57BL6/J male mice on chow diet were used. Mice were injected intraperitoneally with LNA-modified ASOs targeting *Gm15663* or nontargeting control diluted in saline starting at 6 weeks of age for 4 weeks (30 mg/kg, twice a week). ASO-CTL: GACTATACGCGCAATA, ASO-*Gm15663*: AGAGGGTTGCATTAGC. ASOs were synthesized by Integrated DNA Technologies. The experiment was performed with the approval of the IACUC at Joslin Diabetes Center. Dual-energy x-ray absorptiometry scanning was performed at Joslin Animal Physiology Core. Oil Red O staining was performed by Rodent Histopathology Core at Harvard Medical School. Triglyceride levels in the liver were measured using Pointe Scientific Triglycerides Liquid Reagents (T7532, Pointe Scientific). ALT levels were measured using ALT Activity Assay Kit (MAK052, Sigma-Aldrich).

### CRISPR-Cas9 and CRISPR-activation

Mice with liver-specific Cas9 overexpression were generated by crossing Albumin-Cre (Alb-Cre) mice (#003574, The Jackson Laboratory) with Rosa26-LSL-Cas9 knockin mice (#024857, The Jackson Laboratory). For CRISPR-activation, mice with liver-specific dCas9 (nuclease-deactivated)–SunTag-p65-HSF1 (SPH) overexpression were generated through crossing Alb-Cre mice with pCAG-LSL-dCas9-SPH knockin mice (#031645, The Jackson Laboratory). Primary hepatocytes were isolated from these mice followed by transfection with tracrRNA and crRNA targeting specific chromatin regions. For each well in a 24-well plate, 12.5 pmol of tracrRNA and 12.5 pmol of crRNA were transfected using 1.5 μl of Lipofectamine MessengerMax (Thermo Fisher Scientific). tracrRNA and crRNA were synthesized by Horizon Discovery.

crRNAs targeting enhancer 1 in the TAD of *Gm15663*/*Tmtc2* were as follows: Enh1-A (TAACGCATGCTTGAATCCAA), Enh1-B (TATTATGAAGGTACCTGATT), Enh1-C (GAGGTTATAGTGACATCAGT), Enh1-D (CTTTAACGGATTCTCAGAAT), and Enh1-E (ACCGAGCAACAACACACATT).

crRNAs targeting enhancer 2 in the TAD of *Gm15663*/*Tmtc2* were as follows: Enh2-A (TTAGAGTTTTGGAGTACCAT), Enh2-B (ATTTTAAGTCGGGTCAGCAA), Enh2-C (TCAAGGTGCCATTTAGGGTC), Enh2-D (TATTATCTGGTTAGCAGTGT), Enh2-E (ACTTTATTTGTAGTATTATC), and Enh2-F (TAATACTACAAATAAAGTCA).

crRNAs targeting the first exon of *Gm15663* were as follows: Gm-2 (GAGAGTGGGGCCAGAGTACC), Gm-1 (GTGGGGCCAGAGTACCAGGC), Gm-5 (GCTTTCCAGAGTCAAAAAGA), Gm-4 (CAGAGTCAAAAAGAAGGTGA), and Gm-3 (AGGAAGATGAAGAAGAGAGT).

crRNAs targeting the upstream of the first exon of *Gm15663* were as follows: Up-3 (TGTCTACCCCCACTGGCTGG), Up-2 (CCCTGTCTACCCCCACTGGC), and Up-1 (TCCTCCCTGTCTACCCCCAC). crRNAs targeting the downstream of the first exon of *Gm15663* were as follows: Down-1 (ATGTGTGTTCAACCTCCAAC), Down-3 (TCAAAATATTATGAGCACGT), and Down-2 (AATCACAATTTGCCTGTTGG).

### Immunoblotting

Proteins were extracted using radioimmunoprecipitation assay buffer (Sigma-Aldrich) supplemented with protease and phosphatase inhibitors. Protein lysates were separated from insoluble material by centrifugation at 11,000 rpm for 10 min at 4°C. Proteins in the lysate were then separated in NuPAGE 4 to 12% bis-tris Midi gels (Thermo Fisher Scientific) with MOPS running buffer. Proteins were transferred to polyvinylidene difluoride membranes (Thermo Fisher Scientific) and blotted with primary antibodies and the corresponding secondary antibodies. Primary antibodies for immunoblotting were as follows: Vinculin: MAB3574 (Millipore-Sigma); Firefly-Luciferase: ab241541 (Abcam); β-tubulin: 2146 (Cell Signaling Technology); TMTC2: HPA026954 (Millipore-Sigma); and PPARγ: sc-7196 (Santa Cruz Biotechnology).

### Plasmid synthesis and transfection

Plasmids were obtained from Addgene or synthesized by GeneUniversal Company. Plasmids were sequenced using Sanger sequencing (Eton Biosciences) or complete plasmid sequencing (MGH CCIB core). Plasmids were transfected to cells using Lipofectamine 3000 Transfection Reagent (Thermo Fisher Scientific). Briefly, cells were seeded to achieve 80% confluence at the time of transfection. For each well in a 12-well plate, 1 μg of plasmids were transfected with 3 μl of Lipofectamine and 2 μl of P3000. For 6- or 24-well plates, the amount of plasmid was adjusted proportionately.

### siRNA synthesis and transfection

siRNA (synthesized by Horizon Discovery) was transfected to cells using Lipofectamine RNAiMAX (Thermo Fisher Scientific). Briefly, cells were seeded to achieve 80% confluence at the time of transfection. For each well in a 12-well plate, 25 to 75 pmol of siRNA were transfected with 5 μl of Lipofectamine RNAiMAX. For 6- or 24-well plates, the amount of siRNA was adjusted proportionately.

### 5′-RACE and complete amplicon sequencing

Total RNA was isolated from mouse liver using TRIzol. RNA with a polyA tail was separated using Dynabeads mRNA Purification Kit (Thermo Fisher Scientific) followed by 5′-RACE using SMARTer RACE kit (Takara Bio). The 5′ end region of *Gm15663* was amplified using a general 5′-RACE primer provided by SMARTer RACE kit and a primer specific to *Gm15663* (sequence: GGCCCTTCCATCCCAATTCA). The size of 5′-RACE PCR product was analyzed using agarose gel electrophoresis. Subsequently, the 5′-RACE PCR product was subjected to complete amplicon sequencing at MGH CCIB core to determine the sequences within the PCR product along with their respective proportions. Reads aligned to *Gm15663* gene region were extracted, and the first nucleotide of the *Gm15663* within the read was identified.

### Precision run-on–qPCR

Precision run-on (PRO) was conducted according to the established protocol (*87*). Briefly, nuclei were isolated from primary hepatocytes under conditions in which transcribing Pol II was paused. Biotin-NTPs (biotin-11–adenosine triphosphate: NEL544001EA, Biotin-11–cytidine triphosphate: NEL542001EA, Biotin-11–guanosine triphosphate: NEL545001EA, Biotin-11–uridine triphosphate: NEL543001EA; Revvity) were then incorporated into nascent RNAs during transcription by Pol II. The nucleic RNA was isolated using TRIzol, and biotin-labeled RNA was extracted using streptavidin beads (Dynabeads M-280 Streptavidin, Thermo Fisher Scientific). The level of *Gm15663* was then measured using qRT-PCR.

### Statistical analysis

Data are presented as means ± SEM. Significance was determined using a two-tailed Student’s *t* test, unless otherwise specified in figure legends. Significance is indicated as *P < 0.05, **P < 0.01, ***P < 0.001, ****P < 0.0001.

### Bioinformatic analysis

#### RNA sequencing

The quality of pair-end sequence .fastq files was assessed using FastQC (version 0.11.3). The alignment of reads was evaluated using STAR (version 2.7.0) and Qualimap (version 2.2.1). All samples met the quality threshold. Reads were subsequently mapped to mm10 cDNA references using Salmon. Systematic quality assessment was performed using MultiQC (version 1.5). These steps were performed on Harvard Medical School O2 cluster computer. The relative expression levels of genes were defined using DEseq2. Genes were classified as significantly regulated if they satisfied both *P* value <0.05 and |log_2_(fold change)| > 0.322. If both genes in a bidirectional gene pair passed through this threshold, they are defined as coregulated gene pair. Raw liver RNA-seq data from fasting/refeeding experiments were downloaded from GSE137385 (male C57BL6/J mice on low-fat diet). Raw liver RNA-seq data from insulin clamp experiments were downloaded from GSE117741 (12 mU kg^−1^ min^−1^ insulin versus saline, 3 hours). For Fig. 5, RNA-seq libraries of *Tmtc2* knockdown and *Gm15663* trans-overexpression were generated and sequenced at Harvard Medical School Biopolymers Facility. The data were deposited in GEO under accession number GSE335839. The TF enrichment analysis on differential expressed genes was performed using ChEA3 (*88*). The coding potential of genes was predicted using CPPred (*89*).

#### ChIP-seq/ATAC-seq

ChIP-seq and ATAC-seq datasets were analyzed using Cistrome platform developed by X. S. Liu laboratory (*57*, *58*), and the reads were mapped to mouse mm10 genome. The quality of these datasets was assessed using these criteria: (i) sequence quality metrics (raw sequence median quality scores and GC contents), (ii) mapping quality assessed by uniquely mapped read ratio, (iii) library complexity evaluated using the PCR bottleneck coefficient according to ENCODE guidelines (https://genome.ucsc.edu/ENCODE/qualityMetrics.html), (iv) ChIP enrichment defined by the presence of a sufficient number of high-confidence peaks (>500 peaks with ≥10-fold enrichment), (v) signal-to-noise ratio measured by the fraction of reads in peaks, and (vi) regulatory relevance assessed by the overlap with deoxyribonuclease I hypersensitive sites among the top 5000 peaks. The bigwig files were visualized using WashU epigenome browser. ChIP-seq and ATAC-seq datasets were as follows: Mouse FOXO1 ChIP-seq on livers in Fig. 2: GSE119713; Mouse RAD21 ChIP-seq on livers in Fig. 2: GSM2790416; Mouse CTCF ChIP-seq on livers in Fig. 2: GSM2790410; Mouse BRD4 ChIP-seq on livers in Fig. 2: GSM2790419; Mouse MED1 ChIP-seq on livers in Fig. 2: GSM2790411; Mouse Pol II ChIP-seq on livers in fig. S3: GSE60578 and GSE35790; Mouse H3K4me3 ChIP-seq on livers in fig. S7: GSE35790; Mouse H3K4me1 ChIP-seq on livers in Fig. 6 and fig. S7: GSM2055359; Mouse P300 ChIP-seq on livers in fig. S7: ENCSR765RPR_2; Mouse H3K27Ac ChIP-seq on livers in fig. S7: GSM2055365; Mouse ATAC-seq on livers in Fig. 2: GSE191030; Mouse ATAC-seq on MoDC in fig. S4: GSM1531750; Mouse ATAC-seq on pDC in fig. S4: GSM1531751; Mouse ATAC-seq on ALP in fig. S4: GSM1635403; Mouse ATAC-seq on neuron in fig. S4: GSM1541964; Mouse ATAC-seq on myotube in fig. S4: GSM2394776; Mouse ATAC-seq on adipose tissue in fig. S4: GSM2816568; Mouse ATAC-seq on intestinal stem cells in fig. S4: GSM2201101.

#### Hi-C analysis

The Hi-C interaction matrix was visualized using HiGlass at a resolution of 5 kb. Briefly, the Hi-C interaction matrix (GSE104129) was converted to .mcool files using hicpro2higlass.sh utility at a resolution of 5 kb within the HiC-Pro. These steps were performed on Harvard Medical School O2 cluster computer. Subsequently, the .mcool file was ingested under the Docker environment provided by HiGlass using “docker exec higlass-container python higlass-server/manage.py ingest_tileset --filename iced.mcool --filetype cooler --datatype matrix”. The data were then viewed using the HiGlass browser (*90*). The virtual 4C was performed on Harvard Medical School O2 cluster computer.
