# Supplementary material for: RNA-independent cis-autoregulatory circuits within bidirectional gene pairs control metabolism
Source: Sci Adv. 2026 Sep 11;12(37):eaei6204. doi: 10.1126/sciadv.aei6204 (PMC13564825; doi:10.1126/sciadv.aei6204)
Supplement: Supplementary file 1 [file sciadv.aei6204_sm.pdf]

Science Advances
AAAS

Supplementary Materials for

RNA-independent cis-autoregulatory circuits within bidirectional gene pairs control metabolism

Xiang-Yu Liu *et al.*

Corresponding author: C. Ronald Kahn, c.ronald.kahn@joslin.harvard.edu

Sci. Adv. **12**, eaei6204 (2026)
DOI: 10.1126/sciadv.aei6204

The PDF file includes:

Figs. S1 to S9
Legends for tables S1 to S4
Legends for movies S1 and S2

Other Supplementary Material for this manuscript includes the following:

Tables S1 to S4
Movies S1 and S2

Figure S1

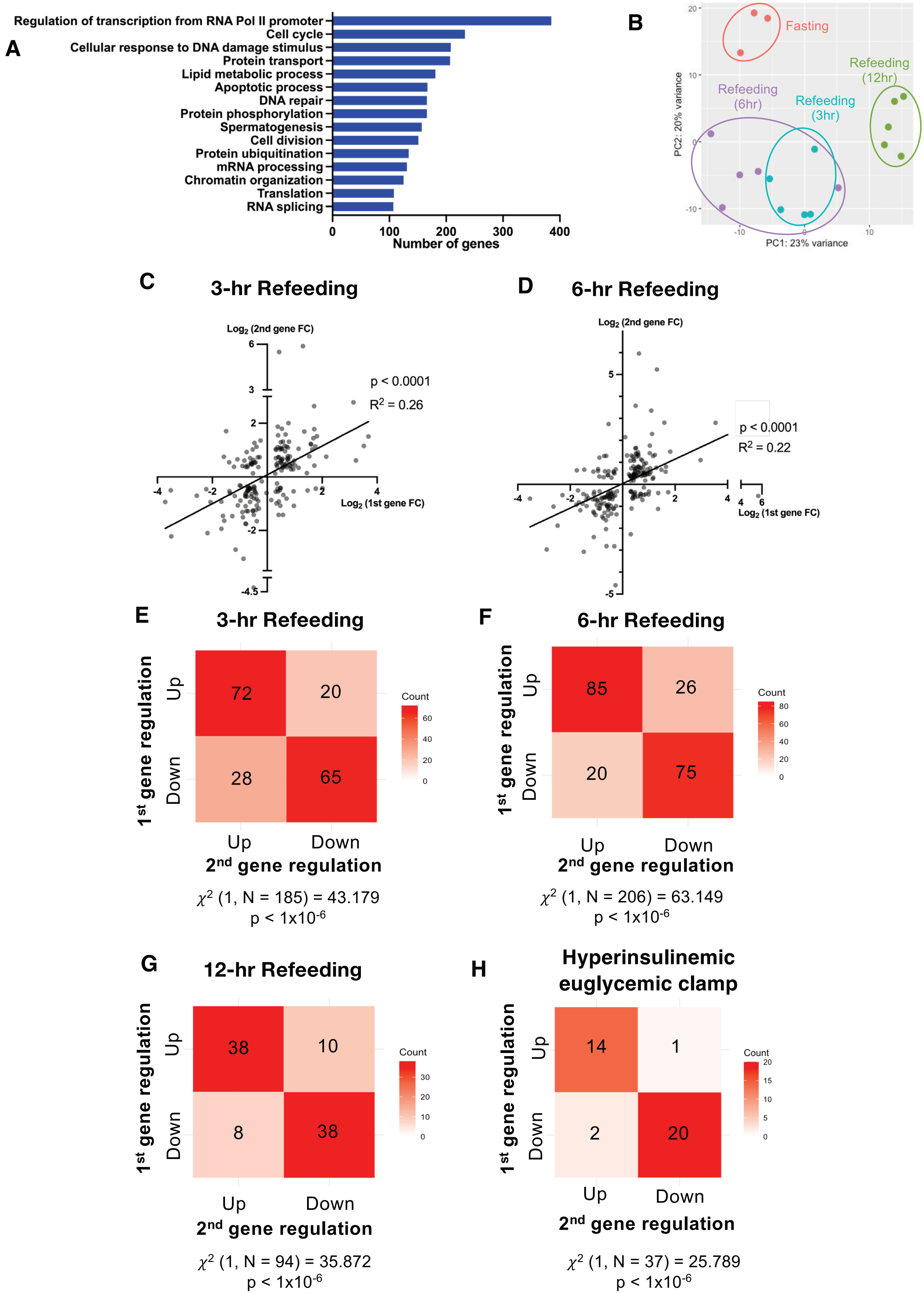
A
Regulation of transcription from RNA Pol II promoter
Cell cycle
Cellular response to DNA damage stimulus
Protein transport
Lipid metabolic process
Apoptotic process
DNA repair
Protein phosphorylation
Spermatogenesis
Cell division
Protein ubiquitination
mRNA processing
Chromatin organization
Translation
RNA splicing
Number of genes
B
Fasting
Refeeding (12hr)
Refeeding (6hr)
Refeeding (3hr)
PC2: 20% variance
PC1: 23% variance
C
3-hr Refeeding
Log2 (2nd gene FC)
Log2 (1st gene FC)
p < 0.0001
R2 = 0.26
D
6-hr Refeeding
Log2 (2nd gene FC)
Log2 (1st gene FC)
p < 0.0001
R2 = 0.22
E
3-hr Refeeding
1st gene regulation
2nd gene regulation
Up
Down
72
20
28
65
Count
χ2 (1, N = 185) = 43.179
p < 1x10-6
F
6-hr Refeeding
1st gene regulation
2nd gene regulation
Up
Down
85
26
20
75
Count
χ2 (1, N = 206) = 63.149
p < 1x10-6
G
12-hr Refeeding
1st gene regulation
2nd gene regulation
Up
Down
38
10
8
38
Count
χ2 (1, N = 94) = 35.872
p < 1x10-6
H
Hyperinsulinemic euglycemic clamp
1st gene regulation
2nd gene regulation
Up
Down
14
1
2
20
Count
χ2 (1, N = 37) = 25.789
p < 1x10-6

Figure S1. Regulation of bidirectional lncRNA/PCG or PCG/PCG pairs in metabolic transitions.

(A) GO pathways enriched in bidirectional gene pairs on mouse genome using DAVID GO analysis (https://david.ncifcrf.gov/). Bidirectional gene pairs are characterized by the transcription start sites (TSSs) of two genes separated by less than 2 kb apart on opposite DNA strands in mm10 genome.

(B) Principal component analysis of transcriptomics in mouse liver. RNA-seq data are from GSE137385 data set (male C57BL6/J mice on low-fat diet)

(C-D) Regulation of bidirectional gene pairs in mouse liver at 3 or 6 hours after refeeding compared to fasting. Linear regression analysis of change on a $\log_2$ scale.

(E-H) Color-coded contingency tables showing relative intensity of coordinated versus discordant regulation during refeeding (E-G), and during hyperinsulinemic euglycemic clamp (H). The degree of coordinated regulation was statistically tested using χ^2 tests.

RNA-seq data of fasting/feeding transition are from the GSE137385 data set (male C57BL6/J mice on low-fat diet, n = 3-5). RNA-seq data of hyperinsulinemic euglycemic clamp are from GSE117741 (liver samples).

Figure S2

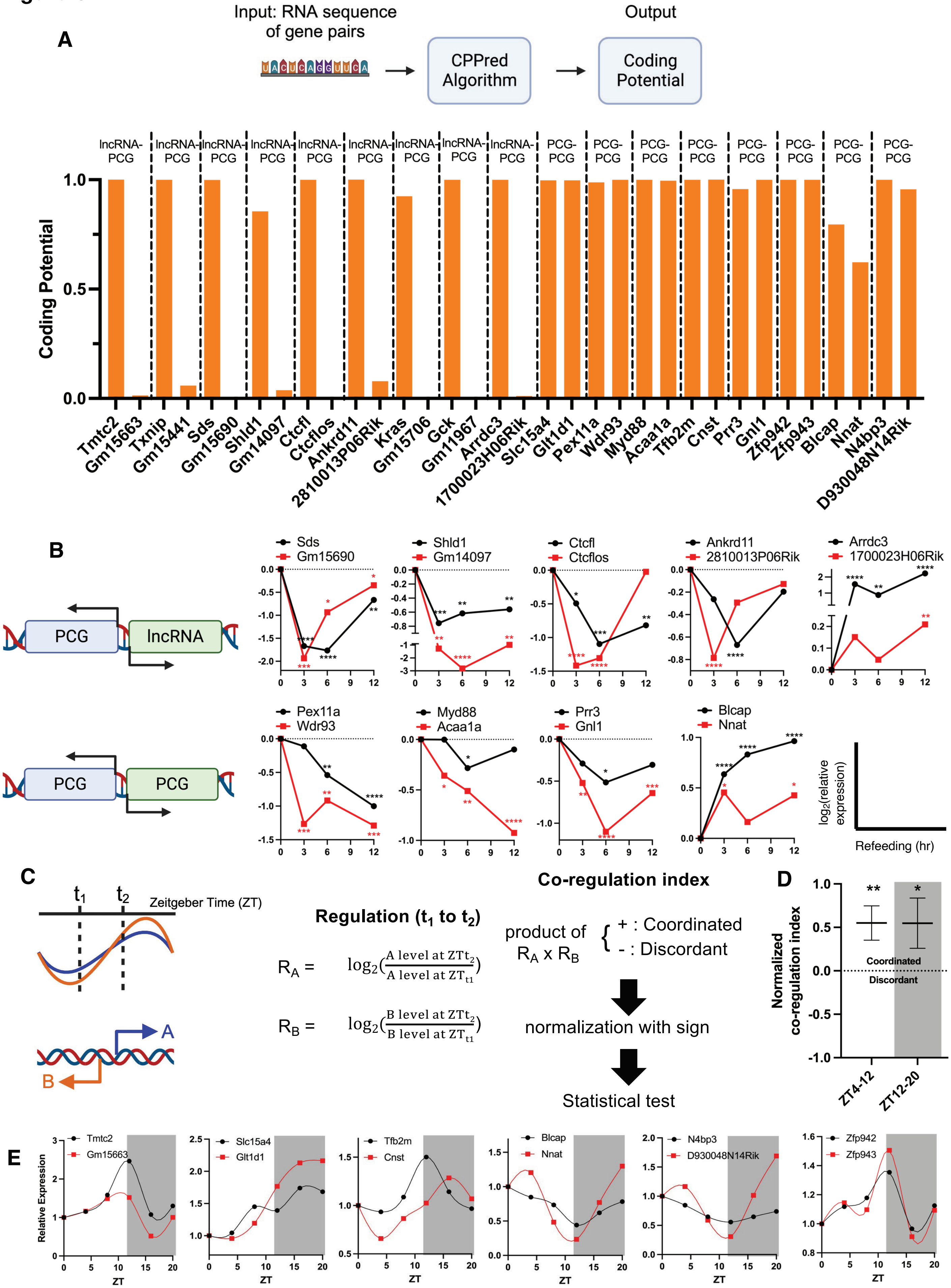

A
Input: RNA sequence of gene pairs
CPPred Algorithm
Output
Coding Potential
lncRNA-PCG
PCG-PCG
Coding Potential
Tmtc2
Gm15663
Txnip
Gm15441
Sds
Gm15690
Shld1
Gm14097
Ctcfl
Ctcflos
Ankrd11
2810013P06Rik
Kras
Gm15706
Gck
Gm11967
Arrdc3
1700023H06Rik
Slc15a4
Glt1d1
Pex11a
Wdr93
Myd88
Acaa1a
Tfb2m
Cnst
Prr3
Gnl1
Zfp942
Zfp943
Blcap
Nnat
N4bp3
D930048N14Rik
B
PCG
lncRNA
PCG
log2(relative expression)
Refeeding (hr)
C
Zeitgeber Time (ZT)
Regulation (t1 to t2)
$R_A = \log_2(\frac{\text{A level at ZT}t_2}{\text{A level at ZT}t_1})$
$R_B = \log_2(\frac{\text{B level at ZT}t_2}{\text{B level at ZT}t_1})$
Co-regulation index
product of $R_A \times R_B$
+ : Coordinated
- : Discordant
normalization with sign
Statistical test
D
Normalized co-regulation index
Coordinated
Discordant
ZT4-12
ZT12-20
E
Relative Expression
ZT

Figure S2. Regulation of bidirectional gene pairs during fasting/feeding and in circadian rhythm.

(A) Gene pairs regulated both during hyperinsulinemic euglycemic clamp and after refeeding, and their coding potential defined by CPPred algorithm. Created in BioRender. Liu, X. (2026) https://BioRender.com/w8fleyq

(B) Transcription regulatory kinetics of lncRNA/PCG and PCG/PCG pairs in mouse liver at fasting and at 3 hours, 6 hours and 12 hours after refeeding. Significance and p value were determined using DESeq2.

(C) Calculation of co-regulation index for bidirectional genes in circadian rhythm. Single-sample t-test was performed for statistical analysis (null hypothesis: two genes are not related during the regulation). Created in BioRender. Liu, X. (2026) https://BioRender.com/w8fleyq

(D) Co-regulation index of bidirectional gene pairs during ZT4-12 and ZT12-20 transitions.

(E) Oscillation of relative expression levels of bidirectional gene pairs in the liver over a circadian cycle. RNA-seq data are from GSE117134 data set, representing samples from 8-12 week-old wild type C57BL6/J mice. Grey: light-off period in a circadian cycle. Data points are plotted on a linear scale.

In all panels above, significance is indicated as *p<0.05, **p<0.01, ***p<0.001, ****p<0.0001. Significance was determined using a two-tailed Student's t test, unless otherwise specified.

Figure S3

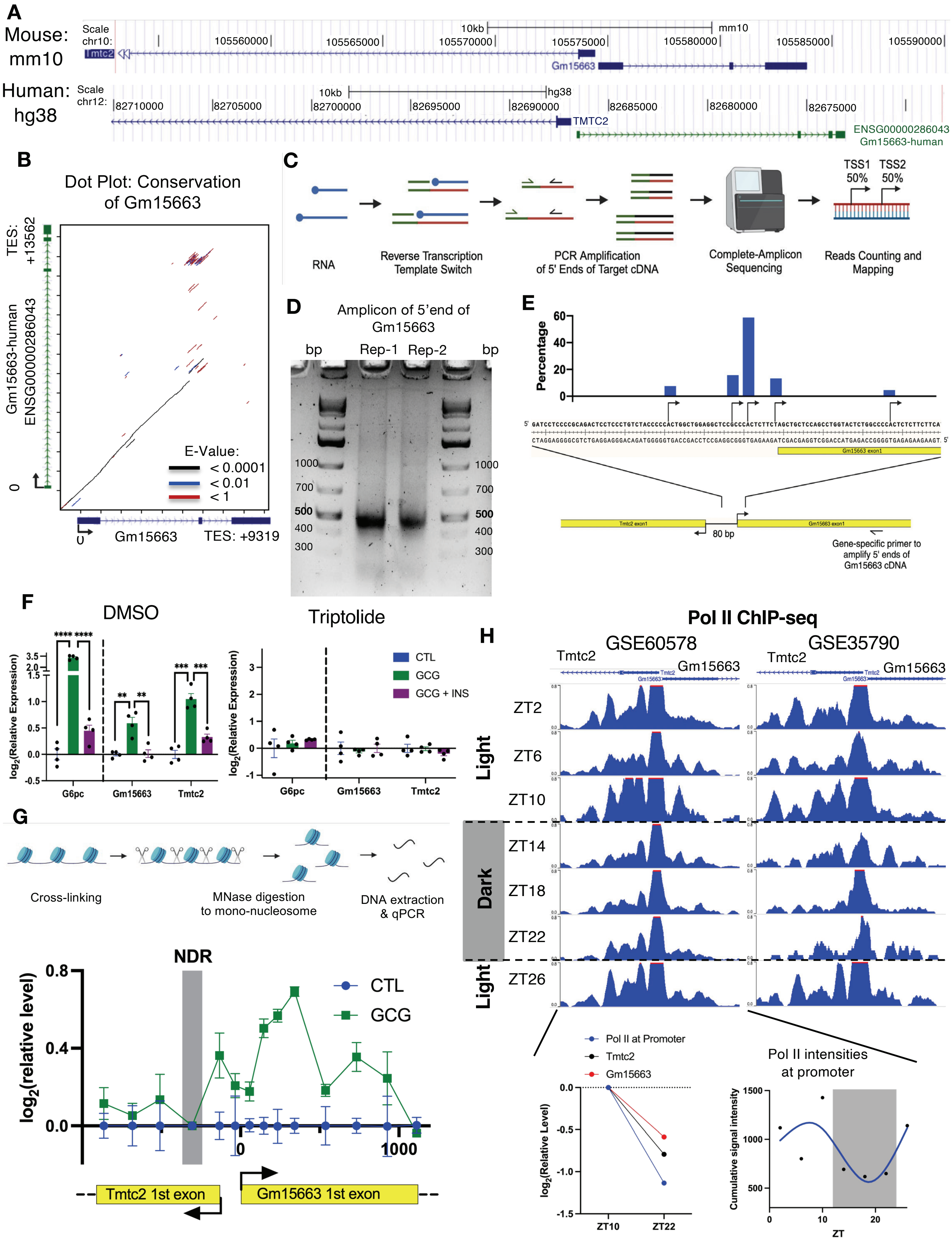

A
Mouse: mm10
Human: hg38
Tmtc2
Gm15663
TMTC2
ENSG00000286043
Gm15663-human
B
Dot Plot: Conservation of Gm15663
Gm15663-human ENSG00000286043
TES: +13562
E-Value:
< 0.0001
< 0.01
< 1
Gm15663
TES: +9319
C
RNA
Reverse Transcription Template Switch
PCR Amplification of 5' Ends of Target cDNA
Complete-Amplicon Sequencing
TSS1 50%
TSS2 50%
Reads Counting and Mapping
D
Amplicon of 5'end of Gm15663
bp
Rep-1
Rep-2
E
Percentage
Tmtc2 exon1
80 bp
Gm15663 exon1
Gene-specific primer to amplify 5' ends of Gm15663 cDNA
F
DMSO
Triptolide
log2(Relative Expression)
G6pc
Gm15663
Tmtc2
CTL
GCG
GCG + INS
G
Cross-linking
MNase digestion to mono-nucleosome
DNA extraction & qPCR
NDR
log2(relative level)
CTL
GCG
Tmtc2 1st exon
Gm15663 1st exon
H
Pol II ChIP-seq
GSE60578
GSE35790
Tmtc2
Gm15663
ZT2
ZT6
ZT10
ZT14
ZT18
ZT22
ZT26
Light
Dark
Light
Pol II at Promoter
Tmtc2
Gm15663
log2(Relative Level)
ZT10
ZT22
Pol II intensities at promoter
Cumulative signal intensity
ZT

Figure S3. Conservation of Gm15663 and its transcriptional regulation.
(A) Annotated locations of Gm15663 and Tmtc2 in mouse (mm10) and human (hg38) genomes.
(B) Dot plot showing the conservation between Gm15663 gene in mouse (mm10) and human (hg38) genomes. The analysis was performed using PLALIGN alignment algorithm.
(C) Schematic showing the characterization of transcription start sites (TSSs) of a gene using 5'- rapid amplification of cDNA ends (5'RACE) followed by complete amplicon sequencing. Created in BioRender. Liu, X. (2026) https://BioRender.com/w8fleyq
(D-E) Gm15663 TSSs in mouse liver defined by 5'RACE and complete amplicon sequencing. Rep: replicates. Created in BioRender. Liu, X. (2026) https://BioRender.com/w8fleyq
(F) Loss of glucagon or insulin regulation of Gm15663/Tmtc2 in mouse primary hepatocytes treated with triptolide. Triptolide: 1 μM, glucagon or insulin: 100 nM. Expression was assessed by qRT-PCR. Data are means ± SEM (n =4).
(G) MNase-qPCR of chromatin isolated from mouse primary hepatocytes treated with glucagon. NDR: nucleosome-depleted region. Data are means ± SEM. Created in BioRender. Liu, X. (2026) https://BioRender.com/w8fleyq
(H) ChIP-seq signals of RNA Pol II at the promoter of Gm15663/Tmtc2 during a circadian cycle. ChIP-seq datasets are from GSE60578 and GSE35790. Lower panels show transcription levels of Gm15663/Tmtc2 and cumulative Pol II ChIP-seq signal intensities around the promoter of Gm15663/Tmtc2, chr10: 105572800-105575687 (mm10). Grey: light-off period in a circadian cycle.

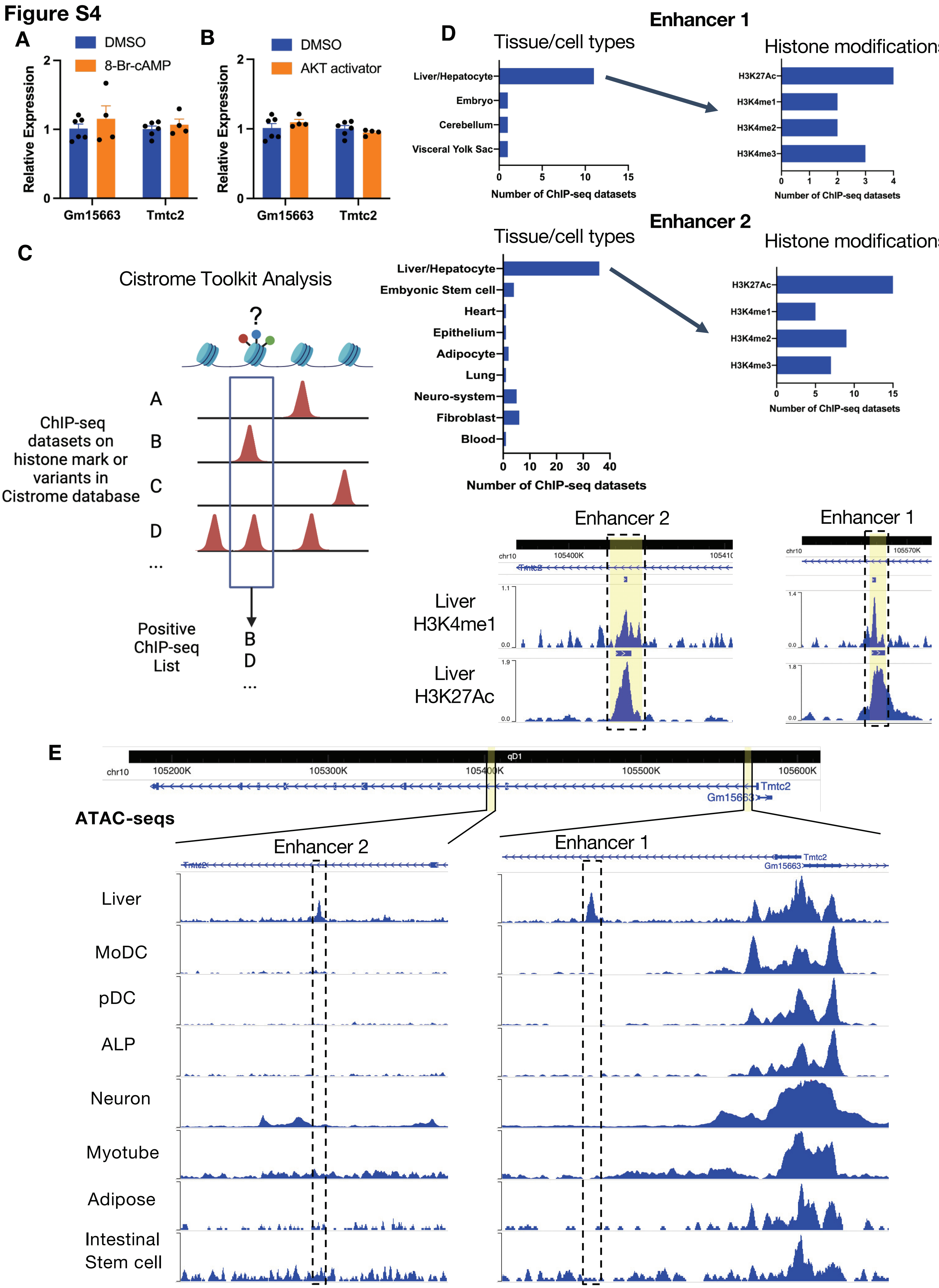

Figure S4
A
DMSO
8-Br-cAMP
Relative Expression
Gm15663
Tmtc2
B
DMSO
AKT activator
Relative Expression
Gm15663
Tmtc2
C
Cistrome Toolkit Analysis
ChIP-seq datasets on histone mark or variants in Cistrome database
Positive ChIP-seq List
D
Enhancer 1
Tissue/cell types
Liver/Hepatocyte
Embryo
Cerebellum
Visceral Yolk Sac
Number of ChIP-seq datasets
Histone modifications
H3K27Ac
H3K4me1
H3K4me2
H3K4me3
Number of ChIP-seq datasets
Enhancer 2
Tissue/cell types
Liver/Hepatocyte
Embyonic Stem cell
Heart
Epithelium
Adipocyte
Lung
Neuro-system
Fibroblast
Blood
Number of ChIP-seq datasets
Histone modifications
H3K27Ac
H3K4me1
H3K4me2
H3K4me3
Number of ChIP-seq datasets
Enhancer 2
Enhancer 1
Liver H3K4me1
Liver H3K27Ac
E
qD1
chr10
105200K
105300K
105400K
105500K
105600K
Tmtc2
Gm15663
ATAC-seqs
Enhancer 2
Enhancer 1
Liver
MoDC
pDC
ALP
Neuron
Myotube
Adipose
Intestinal Stem cell

Figure S4. Gm15663 and Tmtc2 are regulated by FoxO1 and its binding to liver-specific enhancers.

(A) Loss of regulation of Gm15663 and Tmtc2 by 8-Br-cAMP in primary hepatocytes from FoxO1 hepatocyte-specific knockout mice. Treatment with 8-Br-cAMP was at 20 μM for 6 hours. Expression was assessed by qRT-PCR. Data are means ± SEM (n = 4-6).

(B) Loss of regulation of Gm15663 and Tmtc2 by AKT chemical activator SC79 in primary hepatocytes from FoxO1 hepatocyte-specific knockout mice. Treatment with SC79 was at 10 μM for 6 hours. Expression was assessed by qRT-PCR. Data are means ± SEM (n = 4-6).

(C-D) Distribution of histone posttranslational modifications (PTMs) ChIP-seqs which detected signals in Enh1 or Enh2 regions from different tissues in Cistrome database. In (C), A–D schematically represent individual ChIP-seq datasets with or without peak signals at the region of interest. Representative H3K4me1 (GSM1087079) and H3K27Ac (GSM2136888) ChIP-seq signals were shown at regions of Enh1 and Enh2. Created in BioRender. Liu, X. (2026) https://BioRender.com/w8fleyq

(E) ATAC-seq data on different tissues from mice. See the Methods section for the origins of ATAC-seq datasets. MoDC: monocyte-derived dendritic cells. pDC: plasmacytoid-derived dendritic cells. ALP: all-lymphoid progenitors.

Figure S5

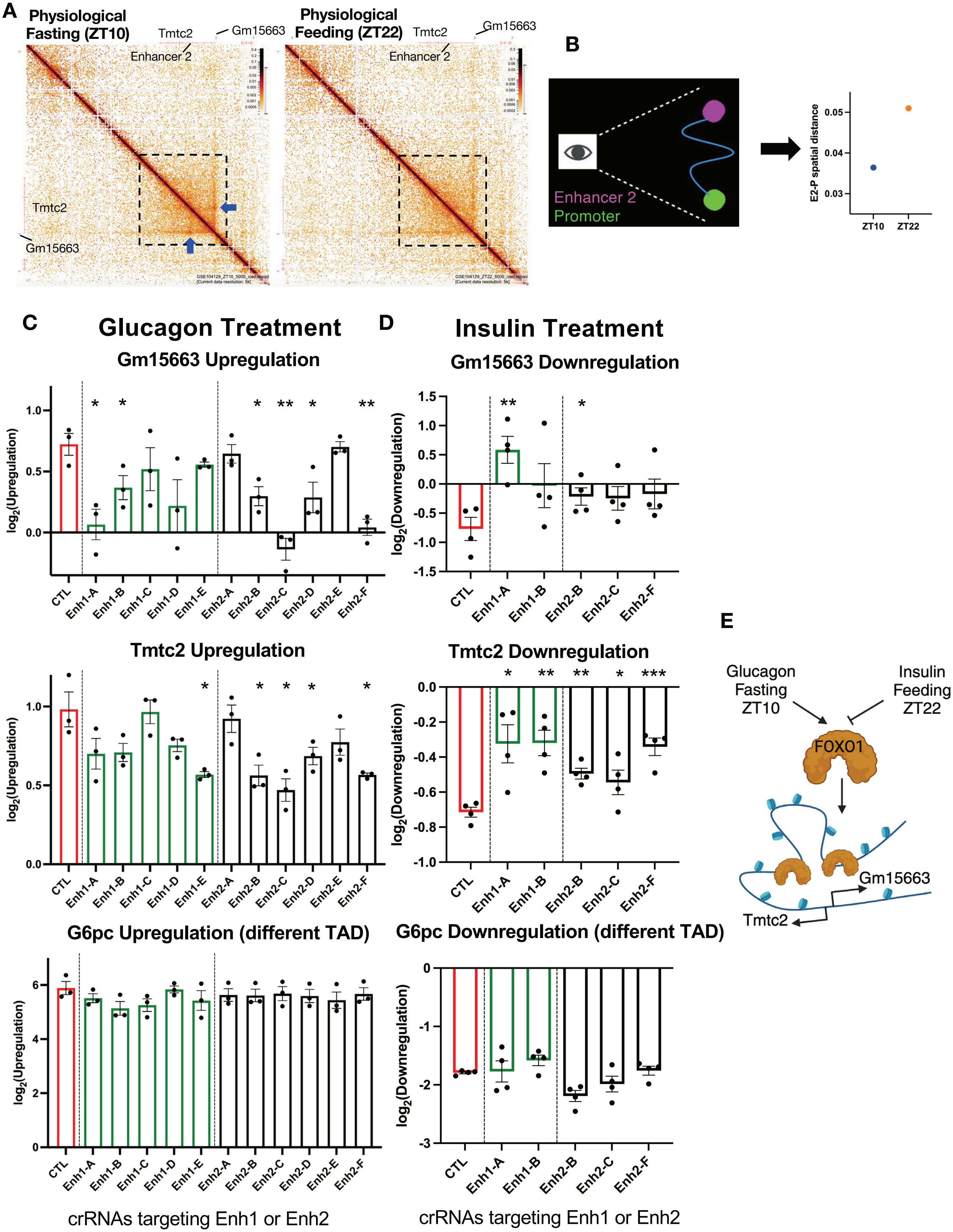
A
Physiological Fasting (ZT10)
Tmtc2
Gm15663
Enhancer 2
Physiological Feeding (ZT22)
B
Enhancer 2
Promoter
E2-P spatial distance
0.05
0.04
0.03
ZT10
ZT22
C
Glucagon Treatment
Gm15663 Upregulation
log2(Upregulation)
Tmtc2 Upregulation
G6pc Upregulation (different TAD)
CTL
Enh1-A
Enh1-B
Enh1-C
Enh1-D
Enh1-E
Enh2-A
Enh2-B
Enh2-C
Enh2-D
Enh2-E
Enh2-F
crRNAs targeting Enh1 or Enh2
D
Insulin Treatment
Gm15663 Downregulation
log2(Downregulation)
Tmtc2 Downregulation
G6pc Downregulation (different TAD)
crRNAs targeting Enh1 or Enh2
E
Glucagon Fasting ZT10
Insulin Feeding ZT22
FOXO1
Gm15663
Tmtc2

Figure S5. Chromatin topology of promoter and enhancers of Gm15663 and Tmtc2.
(A) Hi-C contact maps at ZT10 (fasting) and ZT22 (feeding) in the liver at 5kb resolution. Hi-C datasets were from GSE104129 (male C57BL6/J mice). Blue arrows are showing the interaction between Enh2 and promoter region of Gm15663/Tmtc2.
(B) Distance between Enh2 and promoter of Gm15663/Tmtc2 based on simulated chromatin spatial organization using FLAMINGO.
(C) Regulation of Gm15663, Tmtc2, and G6pc by glucagon after enhancer disruption using CRISPR-Cas9 in primary hepatocytes. Expression was assessed by qRT-PCR. Data are means $\pm$ SEM (n = 3).
(D) Regulation of Gm15663, Tmtc2, and G6pc by insulin after enhancer disruption using CRISPR-Cas9 in primary hepatocytes. Expression was assessed by qRT-PCR. Data are means $\pm$ SEM (n = 4).
(E) FoxO1 interacts with promoter-distal enhancers, and the topological loop between these enhancers and promoter upregulates Gm15663 and Tmtc2. Created in BioRender. Liu, X. (2026) https://BioRender.com/w8fleyq

Figure S6

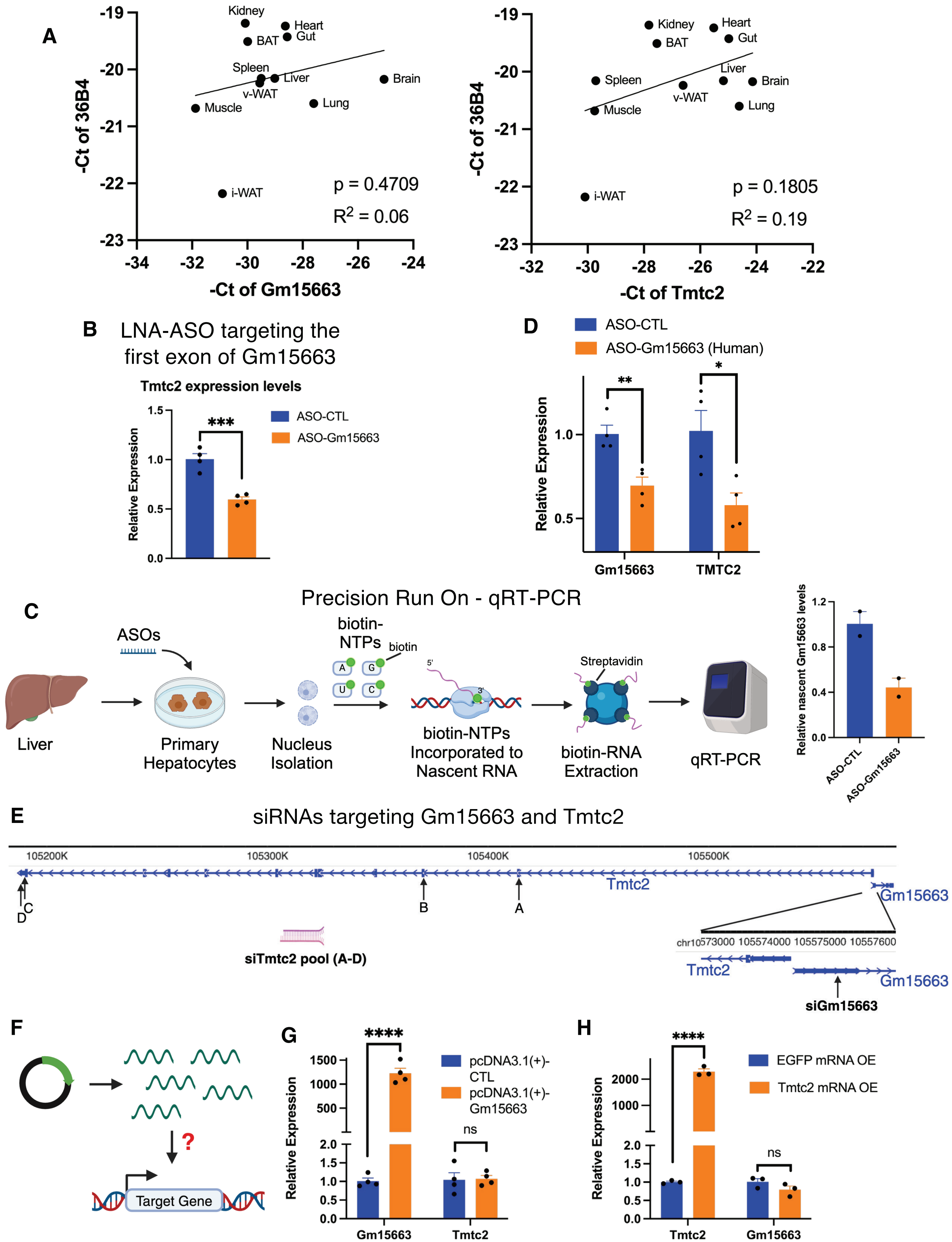

A
-Ct of 36B4
-Ct of Gm15663
Kidney
Heart
Gut
BAT
Spleen
Liver
Brain
v-WAT
Lung
Muscle
i-WAT
p = 0.4709
R^2 = 0.06
-Ct of Tmtc2
p = 0.1805
R^2 = 0.19
B
LNA-ASO targeting the first exon of Gm15663
Tmtc2 expression levels
Relative Expression
ASO-CTL
ASO-Gm15663
***
D
ASO-CTL
ASO-Gm15663 (Human)
**
*
Gm15663
TMTC2
C
Precision Run On - qRT-PCR
ASOs
biotin-NTPs
biotin
Streptavidin
Liver
Primary Hepatocytes
Nucleus Isolation
biotin-NTPs Incorporated to Nascent RNA
biotin-RNA Extraction
qRT-PCR
Relative nascent Gm15663 levels
ASO-CTL
ASO-Gm15663
E
siRNAs targeting Gm15663 and Tmtc2
105200K
105300K
105400K
105500K
Tmtc2
Gm15663
siTmtc2 pool (A-D)
chr10573000 105574000 105575000 10557600
siGm15663
F
Target Gene
G
pcDNA3.1(+)-CTL
pcDNA3.1(+)-Gm15663
****
ns
H
EGFP mRNA OE
Tmtc2 mRNA OE

Figure S6. Cis-regulation between Gm15663 and Tmtc2.
(A) Relative expression levels of Gm15663, Tmtc2 and 36B4 across different tissues in mice. Expression was assessed by qRT-PCR. The same amount of RNA was used for RT reaction across all tissue samples. v-WAT: visceral white adipose tissue. i-WAT: inguinal adipose tissue. BAT: brown adipose tissue. Linear regression analysis of negative Ct values.
(B) Relative transcriptional level of Tmtc2 in mouse primary hepatocytes treated with ASO targeting the first exon of Gm15663. Gapmer-ASOs were modified by locked nucleic acid (LNA) at both ends. The targeting site of ASO-Gm15663 is the same as Gm-ex1-3 in Fig. 3C. Expression was assessed by qRT-PCR. (n =4).
(C) Schematic showing the measurement of the transcription of Gm15663 using PRO-qPCR in mouse primary hepatocytes treated with ASOs. The ASOs were the same as (B). Created in BioRender. Liu, X. (2026) https://BioRender.com/wauaqxo
(D) Relative transcriptional level of Gm15663/TMTC2 in HepG2 hepatocytes treated with ASO targeting the first exon of Gm15663. Gapmer-ASOs were modified by locked nucleic acid (LNA) at both ends. Expression was assessed by qRT-PCR. (n =4)
(E) Schematic showing target sites of siRNAs for Gm15663 and Tmtc2. Created in BioRender. Liu, X. (2026) https://BioRender.com/wauaqxo
(F) Schematic showing trans-overexpression of RNA transcripts using plasmids. Created in BioRender. Liu, X. (2026) https://BioRender.com/wauaqxo
(G) Relative transcriptional level of Tmtc2 in primary hepatocytes treated with plasmids to induce the trans-overexpression of Gm15663. Expression was assessed by qRT-PCR. (n =4).
(H) Relative transcriptional level of Gm15663 in primary hepatocytes treated with plasmids to induce the trans-overexpression of Tmtc2. The cDNA of Tmtc2 containing its 5'UTR, ORF and 3'UTR is inserted to pEGFP-N1-FLAG backbone plasmid. Expression was assessed by qRT-PCR. (n =3).
Data are means ± SEM. In all panels above, significance is indicated as *p<0.05, **p<0.01, ***p<0.001, ****p<0.0001. Significance was determined using a two-tailed Student's t test, unless otherwise specified.

Figure S7

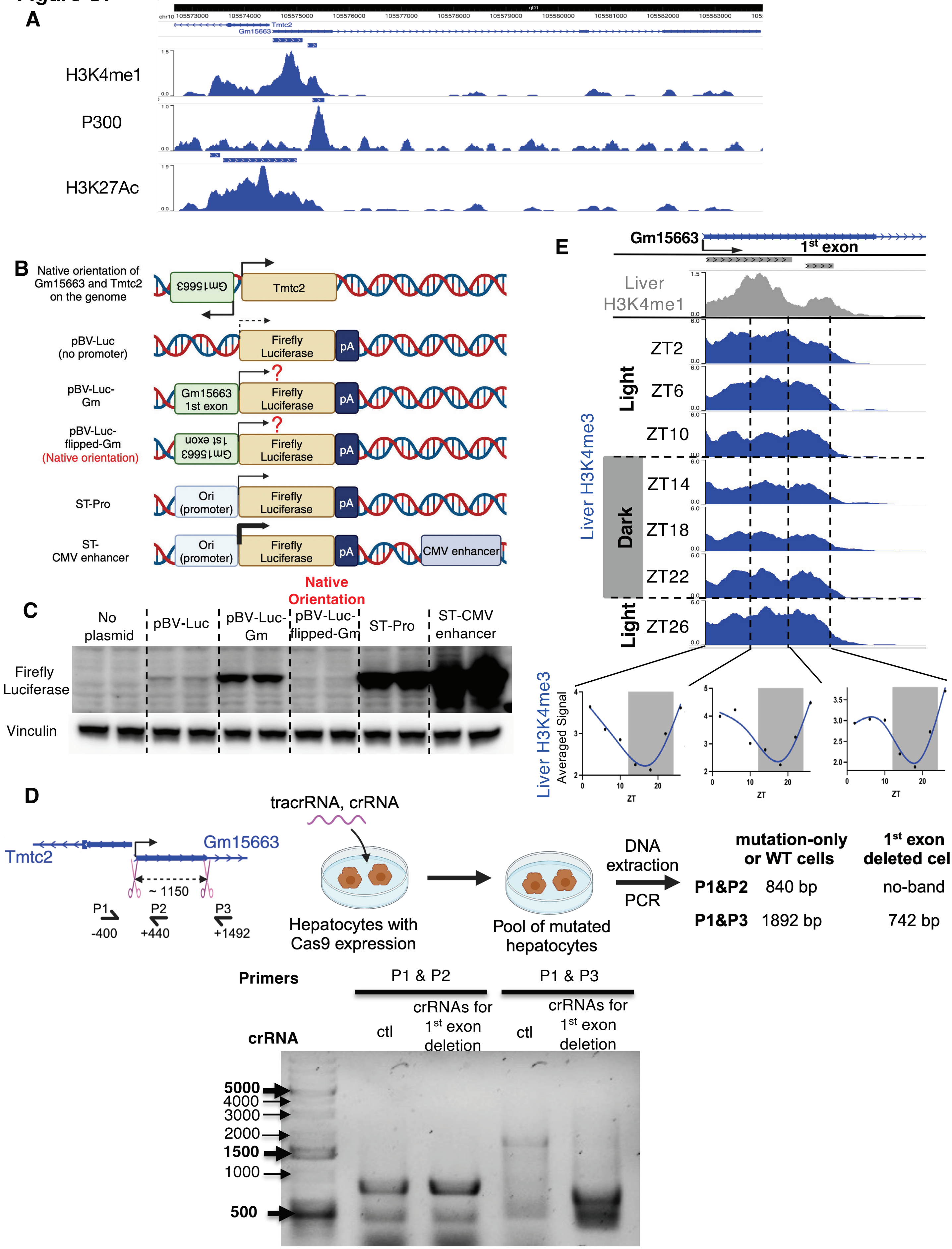

A
H3K4me1
P300
H3K27Ac
B
Native orientation of Gm15663 and Tmtc2 on the genome
Gm15663
Tmtc2
pBV-Luc (no promoter)
Firefly Luciferase
pA
pBV-Luc-Gm
Gm15663 1st exon
pBV-Luc-flipped-Gm (Native orientation)
ST-Pro
Ori (promoter)
ST-CMV enhancer
CMV enhancer
C
No plasmid
pBV-Luc
pBV-Luc-Gm
Native Orientation
pBV-Luc-flipped-Gm
ST-Pro
ST-CMV enhancer
Firefly Luciferase
Vinculin
D
Tmtc2
Gm15663
~ 1150
P1
P2
P3
-400
+440
+1492
tracrRNA, crRNA
Hepatocytes with Cas9 expression
Pool of mutated hepatocytes
DNA extraction
PCR
mutation-only or WT cells
1st exon deleted cells
P1&P2 840 bp no-band
P1&P3 1892 bp 742 bp
Primers
P1 & P2
P1 & P3
crRNA
ctl
crRNAs for 1st exon deletion
5000
4000
3000
2000
1500
1000
500
E
Gm15663
1st exon
Liver H3K4me1
Liver H3K4me3
Light
Dark
ZT2
ZT6
ZT10
ZT14
ZT18
ZT22
ZT26
Averaged Signal
ZT

Figure S7. The first exon region of Gm15663 functions as a promoter-proximal enhancer for Tmtc2.

(A) H3K4me1, p300 and H3K27Ac ChIP-seq data in the region close to shared Gm15663/Tmtc2 promoter in mouse liver. See the Methods section for the origins of ChIP-seq datasets.

(B) Schematic showing construction of plasmids for promoter activity assay. pA: polyadenylation signal. Created in BioRender. Liu, X. (2026) https://BioRender.com/wauaqxo

(C) Reporter assay of promoter activity of the first exon of Gm15663. The level of firefly luciferase was assessed by immunoblot with vinculin as the reference. AML12 hepatocytes were used in this experiment.

(D) Verification of the knockout of Gm15663 first exon by PCR and electrophoresis. Primer 1 (P1): AGGGCTGGACAGAGCGAGGA; P2: ACTCTCTCCTAAACGCTGCTTCCCTG; P3: ATTTACATGGAAACTCACCCAACCAAAGT. Created in BioRender. Liu, X. (2026) https://BioRender.com/wauaqxo

(E) Cumulative H3K4me3 ChIP-seq signal intensity of the Gm15663-first-exon enhancer. Grey: light-off period in a circadian cycle.

Figure S8

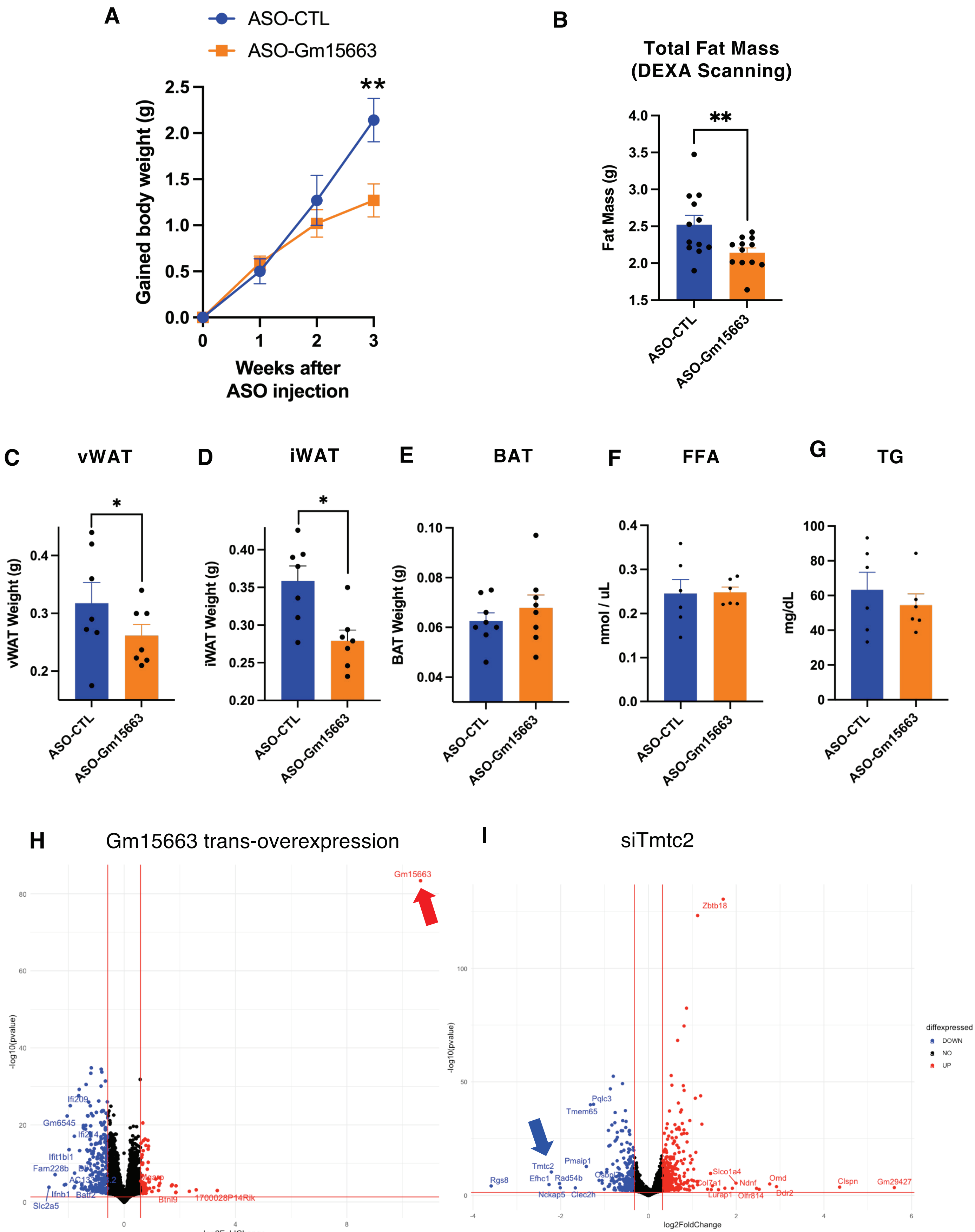
A
ASO-CTL
ASO-Gm15663
Gained body weight (g)
Weeks after ASO injection
B
Total Fat Mass (DEXA Scanning)
Fat Mass (g)
C
vWAT
vWAT Weight (g)
D
iWAT
iWAT Weight (g)
E
BAT
BAT Weight (g)
F
FFA
nmol / uL
G
TG
mg/dL
H
Gm15663 trans-overexpression
I
siTmtc2
-log10(pvalue)
log2FoldChange
diffexpressed
DOWN
NO
UP

Figure S8. The physiological role of Gm15663 in hepatic metabolism.
(A) Body weight gain of mice after the treatment with ASO-CTL or ASO-Gm15663.
(B) Analysis of total fat mass of mice using dual-energy X-ray absorptiometry (DEXA) scanning (n = 12).
(C-E) Weights of vWAT (C), iWAT (D), and BAT (E) of mice treated with ASO-CTL or ASO-Gm15663. (n = 7-8). v-WAT: visceral white adipose tissue (epididymal). i-WAT: inguinal adipose tissue. BAT: brown adipose tissue.
(F-G) Levels of circulating free fatty acid (FFA) and triglyceride (TG) of mice treated with ASO-CTL or ASO-Gm15663. (n = 6).
(H) Volcano plot showing gene expression in hepatocytes with Gm15663 trans-overexpression using plasmids. Red arrow indicates increased Gm15663 level.
(I) Volcano plot showing gene expression in hepatocytes with Tmtc2 knockdown using siRNA. Blue arrow indicates reduced Tmtc2 level.
Data are means ± SEM. In all panels above, significance is indicated as *p<0.05, **p<0.01, ***p<0.001, ****p<0.0001. Significance was determined using a two-tailed Student's t test, unless otherwise specified.

Figure S9

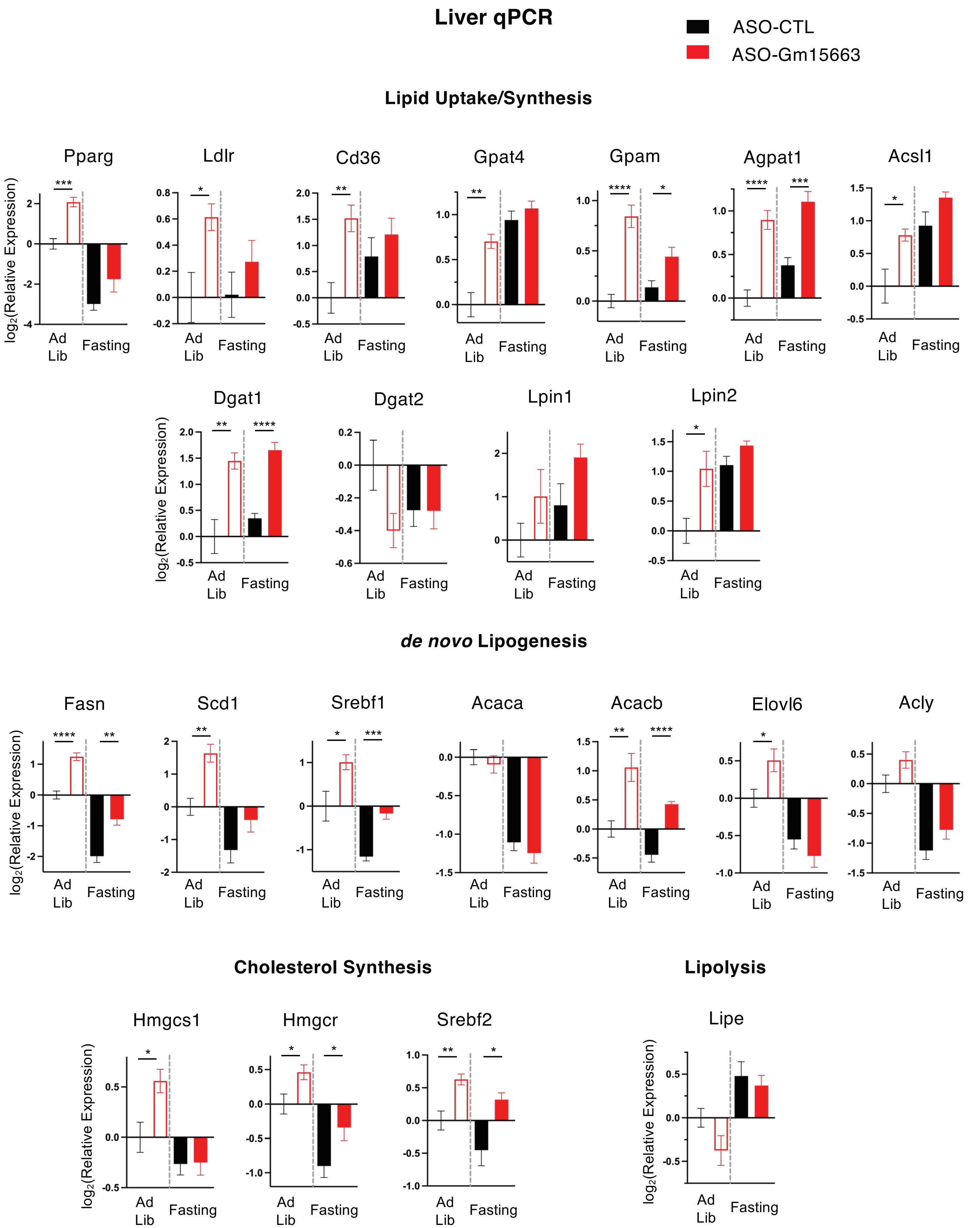

Liver qPCR
ASO-CTL
ASO-Gm15663
Lipid Uptake/Synthesis
Pparg
Ldlr
Cd36
Gpat4
Gpam
Agpat1
Acsl1
Dgat1
Dgat2
Lpin1
Lpin2
de novo Lipogenesis
Fasn
Scd1
Srebf1
Acaca
Acacb
Elovl6
Acly
Cholesterol Synthesis
Hmgcs1
Hmgcr
Srebf2
Lipolysis
Lipe
log2(Relative Expression)
Ad Lib
Fasting

Figure S9. ASO-mediated inhibition of Gm15663 alters hepatic lipid metabolic gene programs.

Relative expression levels of genes involved in lipid uptake/synthesis, *de novo* lipogenesis, cholesterol synthesis, and lipolysis in livers of mice treated with ASO-CTL or ASO-Gm15663 under ad libitum (Ad Lib) or after overnight fasting conditions (n = 6). Expression was assessed by qRT-PCR. Data are means ± SEM.

In all panels above, significance is indicated as *p<0.05, **p<0.01, ***p<0.001, ****p<0.0001. Significance was determined using a two-tailed Student's t test, unless otherwise specified.

Supplementary Tables

Table S1. Coordinately regulated bidirectional gene pairs during fasting and 3-hour refeeding.

Table S2. Coordinately regulated bidirectional gene pairs during fasting and 6-hour refeeding.

Table S3. Coordinately regulated bidirectional gene pairs during fasting and 12-hour refeeding.

Table S4. Coordinately regulated bidirectional gene pairs during insulin clamp.

Supplementary Movies

Movie S1. Simulated 3D chromatin architecture of the Gm15663/Tmtc2 TAD at ZT10.

Movie S2. Simulated 3D chromatin architecture of the Gm15663/Tmtc2 TAD at ZT22.
